# Cell cycle dysregulation: a central hub in colitis-associated colorectal carcinogenesis

**DOI:** 10.3389/fimmu.2026.1775221

**Published:** 2026-07-06

**Authors:** Yuanjie Fu, Ying Wang, Shunjing Wu, Yi Ying, Junxin Li, Yanghuan Ou, Yinying Wang, Li Li, Yueying Wu, Jinyuan Yan, Zhongshan Yang

**Affiliations:** 1 Yunnan Provincial Key Laboratory of Integrated Traditional Chinese and Western Medicine for Chronic Disease in Prevention and Treatment, Yunnan University of Traditional Chinese Medicine, Kunming, Yunnan, China; 2 Yunnan Provincial Department of Education, Engineering Research Center of Classic Formula Regulate Immunity in Chronic Disease Prevention and Treatment, Kunming, Yunnan, China; 3 Artemisinin Research Center, and Institute of Chinese Materia Medica, China Academy of Chinese Medical Sciences, Beijing, China; 4 Center Laboratory of the Second Hospital Affiliated, Kunming Medical University, Kunming, China

**Keywords:** cell cycle, colorectal “inflammation-cancer” transformation, cyclin-dependent kinase inhibitors, Hippo–YAP pathway, oxidative stress

## Abstract

The process of developing from inflammatory bowel disease (IBD) to colorectal cancer (CRC) depends on the dysregulation of the core cell cycle network. Single-cell omics studies have revealed a significant difference: malignant colorectal cancer cells are mainly in the G2/M phase, while epithelial cells in inflammatory bowel disease (IBD) exhibit G1 phase arrest or excessive proliferation - this indicates that during chronic inflammation, the original cell cycle of precancerous cells has already become dysregulated. Among the key signaling pathways, such as Rb–E2F, NF-κB, JAK–STAT and Hippo–YAP, jointly regulate the cell cycle system. Including cyclins D1, A2 and B1 and their cyclin-dependent kinases (CDKs), as well as cyclin-dependent kinase inhibitors (such as p21, p27, p57), jointly regulate intestinal tumor cells and tumor immune escape. Therefore, this review elaborates on the key interactions between ROS and the cell cycle/cyclin/CDK axis, and explains in detail how oxidative stress and DNA damage disrupt cell cycle checkpoints, thereby driving the growth, invasion, metastasis and immune escape of intestinal tumors. In conclusion, for the regulation of the cell cycle during the “inflammation-cancer” transformation process in colorectal cancer, new targets and reasonable combination strategies have been proposed.

## 1 Introduction

According to the 2024 statistical forecast report released by the International Agency for Research on Cancer, there were approximately 20 million new cancer cases worldwide in 2022. Among them, colorectal cancer (CRC) was the second leading cause of cancer-related deaths, accounting for 9.6% of all new cases and 9.3% of total cancer deaths (1). This trend is also supported by recent statistics from the American Cancer Society, which estimates that in 2026, approximately 158,850 people will be diagnosed with CRC and about 55,230 will die from the disease in the United States (2). Common clinical manifestations of CRC include bloody stools and abdominal pain. In recent years, advancements in diagnosis and treatment methods - such as colectomy, adjuvant radiotherapy and chemotherapy, and immunotherapy - have improved treatment outcomes, prolonged patients’ survival, and reduced the mortality rate of CRC (3).

However, an analysis of the mortality rates of 475,771 CRC patients revealed that disease progression remains the leading cause of death within five years after diagnosis. This worrying trend highlights the urgent need for improvement measures. For example: reducing risk factors, strengthening early prevention and screening, deepening understanding of the tumor occurrence and development mechanism, and promoting precise anti-cancer therapies (4).

The pathogenesis of CRC is the result of the combined effects of genetic instability, dysregulation of signaling pathways, DNA damage caused by oxidative stress, and chronic inflammation (5–9). The risk factors for CRC include genetic factors (such as mutations in APC, KRAS, and BRAF), dietary and lifestyle influences, and disease factors such as inflammatory bowel disease (IBD) and obesity, etc. (10–14).

The assessment of risk factors for CRC indicates that the cumulative inflammatory burden is a key factor contributing to the occurrence of CRC (15). In patients with IBD, the risk of developing CRC is 2 to 3 times higher than that of the general population (16, 17). Based on the results of two large-scale population-based studies, the high-risk subgroup characteristics for a significantly increased risk of colorectal cancer in patients with IBD are: being diagnosed at an age younger than 30, having extensive involvement of the colon, and having a longer disease duration (18, 19). The development of Colitis associated colorectal cancer (CAC) is a complex process involving multiple molecular mechanisms. The specific mechanism is that inflammatory mediators cause genetic mutations and promote genomic instability. At the same time, the inflammatory response also promotes the formation of a favorable tumor microenvironment, thereby accelerating the development of CRC (20–23). Compared with sporadic colorectal cancer, CAC has unique pathological and physiological characteristics and mainly progresses in the sequence of “inflammation - dysplasia - carcinogenesis” (24). The persistent inflammatory environment accelerates the renewal of epithelial cells, triggers oxidative stress and replication stress, and reshapes the internal microenvironment, thereby increasing the risk of CRC (25). During this process, neutrophils and myeloid-derived suppressor cells (MDSCs) play a crucial role. Among them, the activation of neutrophils leads to the production of reactive oxygen species(ROS), which further exacerbates oxidative stress and DNA damage, thereby worsening the intestinal inflammatory state (26), and strongly promotes the recruitment of neutrophils, accelerating the process of CAC (27). However, recent evidence also points to a protective, context-dependent role of neutrophils in colitis-associated cancer (28), highlighting the complex and multifaceted involvement of innate immunity in this process. Moreover, MDSCs proliferate significantly in chronic inflammation and tumor microenvironments. They can inhibit the functions of T cells and other immune cells, thereby promoting tumor development and immune evasion (29).

The occurrence of CAC begins with the persistent disturbance of the epithelial cell’s epigenome by the chronic inflammatory microenvironment. This disturbance systematically reprograms the cell cycle regulatory network through various epigenetic levels such as DNA methylation, histone modification, chromatin remodeling, and non-coding RNA, thereby promoting tumor formation (30, 31).

Regarding DNA methylation, in the UC case, the promoter region of the p16 gene often undergoes abnormally high methylation, resulting in its transcriptional silencing (32). In the CAC case, it was further confirmed that p16 is one of the key genes frequently methylated (33). As a key inhibitor of CDK4/6, the loss of p16’s function would relieve the inhibition of the transition from the G1 phase to the S phase (34). As a key inhibitor of CDK4/6, the loss of p16’s function would relieve the inhibition of the transition from the G1 phase to the S phase. RASSF gene methylation is another major epigenetic mechanism driving CRC, especially through silencing of the tumor−suppressor genes RASSF1A and RASSF2 (35). The most recent and comprehensive evidence shows that RASSF2 promoter hypermethylation is one of the most frequent and clinically significant alterations, occurring in up to 86% of colon cancers, and is associated with worse overall survival (36). Mechanistically, RASSF1A acts as a gatekeeper of the G1/S transition by inhibiting cyclin D1 accumulation and transcriptionally upregulating the CDK inhibitor p21WAF1/CIP1, thereby reinforcing Rb-dependent cell cycle arrest (37–39). Its epigenetic silencing, together with p16 inactivation, removes complementary restraints on G1/S progression, further illustrating how chronic inflammation−driven methylation directly unleashes uncontrolled proliferation during CAC.

Regarding histone modification, a study has shown that in the state of inflammatory bowel disease, the expression level of the histone methyltransferase SETD2 decreases, which exacerbates oxidative stress and thereby promotes colonic inflammation and tumor formation (40). Meanwhile, inflammatory factors such as IL-6 can be catalyzed by the STAT3 signaling pathway. The deposition of the inhibitory mark H3K27me3 on the genes that negatively regulate the cell cycle further reinforces their silenced state (31).

Non-coding RNAs, especially microRNAs (miRNAs), are important epigenetic effectors that link the inflammatory microenvironment with cell cycle regulation. zebrafish model (ImiR-21) with inducible overexpression of miR-21 in the intestine. The results demonstrate that miR-21 can induce CRC or CAC in ImiR-21 through the PI3K/AKT, PDCD4/TNF-α, and IL-6/STAT3 signaling network (41).

The research conducted by Fang Sun et al. clearly indicates that the exogenous overexpression of miR-34a can significantly reduce the mRNA and protein levels of CDK6 in colon cancer cells, and this inhibitory effect directly interferes with the phosphorylation process of retinoblastoma protein (Rb) (42).

Since the transcription of MiR-34a is highly dependent on the functional p53, in IBD-CRC, the mutations or loss of function of p53 directly leads to a significant downregulation of miR-34a expression (43). MiR-34a does not only target CDK6; it also regulates multiple oncogenes such as Cyclin D1, MYC, MET, and BCL2 simultaneously (44, 45).

In conclusion, chronic inflammation is a key factor in the occurrence of CRC. The oxidative stress caused by inflammation serves as the dominant inducer of gene mutations, forming the crucial bridge for the “inflammation-cancer” transformation. The following review will continue to focus on the cell cycle dysregulation triggered by the gene mutations, thereby promoting the malignant transformation of intestinal epithelial cells.

## 2 Chronic intestinal inflammation causes abnormal cell cycle through oxidative stress, thereby promoting the occurrence and development of colorectal cancer

**Figure 1:**
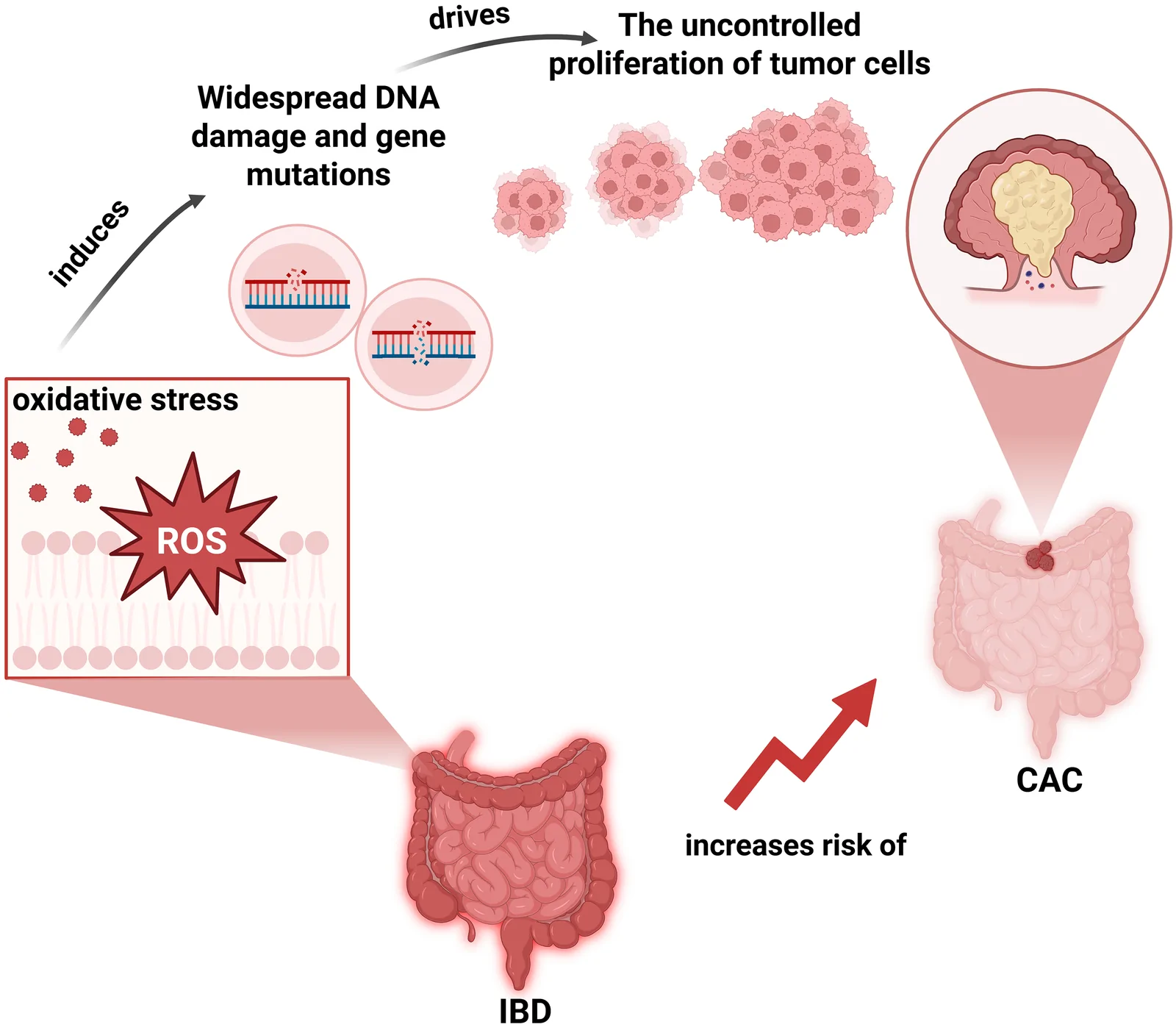
Oxidative stress-induced DNA damage drives tumor formation in inflammatory bowel disease. The DNA damage caused by reactive oxygen species-induced oxidative stress enables the accumulation of mutations and leads to uncontrolled proliferation of tumor cells, which is the key pathogenic mechanism of colon cancer associated with inflammatory bowel disease.

In the state of IBD, continuous release of inflammatory cytokines can cause oxidative stress in cells, thereby promoting the occurrence and development of CRC (46). Among them, the increase in ROS leads to oxidative stress, which causes extensive cell damage (47). ROS can damage membrane lipids and trigger lipid peroxidation reactions. Moreover, ROS can directly damage DNA, inducing various forms of DNA damage such as base oxidation modification, deamination sites formation, single-strand breaks, and potential double-strand breaks, thereby promoting gene mutations and genomic instability (48–50), ultimately resulting in tumor formation as shown in **Figure 1**.

The key function of the cells to prevent the incorrect transmission of genetic material is impaired, directly leading to genomic instability. Two core checkpoints emerge as a result: the G1/S checkpoint and the G2/M checkpoint.

The G1/S checkpoint is a critical node for the cell to decide whether to enter the DNA synthesis phase (S phase). When DNA is damaged due to oxidative stress, the ATM/ATR kinases are activated, which then phosphorylate and stabilize the p53 protein (51, 52). The activated p53 acts as a transcription factor, inducing the expression of p21, which inhibits the activity of the CDK4/6-cyclin D and CDK2-cyclin E complexes, thereby preventing the phosphorylation of RB and keeping the E2F transcription factor inactive, ultimately blocking the cell in the G1 phase for DNA repair or initiating the apoptosis program (53).

The G2/M checkpoint is responsible for ensuring that the cell has completed DNA replication and is free from damage before entering mitosis (M phase). The core regulator of this checkpoint is also p53, but it relies more on the inhibition of the CHK1/CHK2 kinases on the CDC25 phosphatase family. DNA damage activates the ATR-CHK1 pathway, which phosphorylates and inhibits CDC25C, preventing it from activating the CDK1-cyclin B1 complex, thereby blocking the cell in the G2 phase (54).

At the level of cell cycle system regulation, there are fundamental differences between, CAC and sCRC. The root cause lies in the completely different carcinogenic pathways of the two. CAC follows the sequence of “inflammation - dysplasia - cancer”, while sCRC develops through the classic “adenoma - cancer” sequence (24).

It is well known that the most typical feature of sCRC is the early loss of the APC gene, which disrupts the cell cycle regulatory system and leads to uncontrolled proliferation of tumor cells. This is because the APC protein, as a key component of the β-catenin degradation complex, becomes inactive, resulting in abnormal stability of β-catenin in the cytoplasm and its translocation to the nucleus. It then binds to the TCF/LEF transcription factors and continuously activates the expression of downstream target genes such as c-Myc and Cyclin D1 (55). At the same time, activation mutations of oncogenes such as KRAS and BRAF can further promote the expression of Cyclin D1 through the MAPK signaling pathway, and inhibit the functions of CDK inhibitory proteins such as p27, thereby jointly promoting the cell cycle progression (56).

The high expression of Cyclin D1 can trigger tumor cells to pass through the/S phase checkpoint, which is the fundamental cause of the cell cycle disorder in sCRC (57, 58). As the tumor progresses, sCRC accumulates more genetic alterations that affect the cell cycle process. A crucial turning point is the mutation of the TP53 gene. The loss of P53 function allows cells with DNA damage to continue dividing, significantly accelerating chromosomal instability and thereby promoting tumor development (59). In contrast, CAC follows the “inflammation - dysplasia - cancer” sequence (24).

The core feature lies in the continuous remodeling of the cell cycle engine by the chronic inflammatory microenvironment. CAC does not originate from the classic genetic driver sequences, but is driven by the inflammation-injury-repair cycle. In CAC, the inactivation of the p53 tumor suppression pathway is an early and widespread event. The transformation of P53 functional status is a key molecular event driven by inflammation in the process of malignant transformation. Its absence will lead to abnormal cell cycle regulation and impaired cell apoptosis (60). The change in the functional state of P53 is a key molecular event driving inflammatory-driven malignant transformation. In the early stages of CAC, especially before dysplasia, the incidence of TP53 mutations has approached half (60). Simultaneously, research has shown that changes in p53 expression and abnormal levels of inflammatory factors are closely related in patients with UC, and they jointly participate in the pathological process from inflammation to canceration (61). Once a mutation occurs, the function of p53 is not limited to a simple “loss of function”, but many missense mutations also confer new, oncogenic “functions” to the mutant p53 protein, such as: reshaping the inflammatory microenvironment to inhibit anti-tumor immunity, interfering with normal DNA damage response: leading to increased genomic instability and accelerating the accumulation of other oncogenic mutations (62–64).

IBD is a complex chronic inflammatory disorder whose pathogenesis involves a combination of genetic, environmental, microbial, and immune system dysregulation. Cell cycle proteins and their protein kinases play a crucial role in the initiation and progression of IBD. Their dysregulation not only disrupts the homeostasis of the intestinal epithelium but also mediates the inflammatory response and the functions of immune cells.

Multiple studies have shown that the dysregulation of the cell cycle system is associated with intestinal inflammation and impaired barrier integrity. For instance, during intestinal inflammation, macrophages are recruited to the site. One study demonstrated that direct contact between macrophages and crypts activates the Wnt signaling pathway, upregulating targets such as cyclin D1 (65). Furthermore, research by Qian Wang et al. confirmed upregulated expression of cyclin D1 and cyclin B1 in a mouse model of colitis. Although cell proliferation is a reparative response to injury, long - term inflammatory signals (such as IL - 6) strengthen this repair mechanism, leading to excessive epithelial proliferation and dysfunction of the cell layer. Newly proliferated epithelial cells may migrate to the intestinal surface before differentiation and maturation, thus failing to fully support the barrier function. In the context of acute injury, the benefits of rapid repair of the physical barrier brought about by this strong epithelial regenerative capacity outweigh the drawbacks caused by the defects in the chemical barrier (defensins, mucins) (66). However, it cannot be denied that in the context of long - term, low - grade inflammation, this continuous “malformed repair” at the expense of normal cell differentiation results in the epithelial layer being composed of immature and dysfunctional cells for a long time. This state itself represents a form of dysfunction, which will ultimately undermine the long - term stability of the barrier (67). The increase in cyclin D1 indicates a compensatory proliferative response of epithelial cells to chronic inflammatory damage.

Cyclin D1 binds to cyclin-dependent kinase (CDK)4/6, promoting the phosphorylation and inhibition of retinoblastoma protein (Rb), thereby releasing the transcription factor E2F. This process prompts cells to pass through the G1/S checkpoint and enter the DNA synthesis phase leading to excessive and continuous proliferation of intestinal epithelial cells (especially those located at the crypt bottom). The broad-spectrum cell cycle-dependent kinase inhibitor p21 mainly functions by binding to the CDK4/6-cyclin complex and inhibiting its activity. The absence of p21 leads to a significant increase in the activity of CDK4/6 kinase and a change in the state of intestinal stem cells. The research by Liang Xiajiang et al. shows that in mice lacking p21, the reduction of intestinal stem cell compartments, the expansion of Bmi1^+^ cells, the absence of Olfm4^+^/Lgr5^+^ cells, and the decrease in the number of Paneth and Goblet cells in the crypts (68). When treated with palbociclib (a CDK4/6 inhibitor), inhibiting the activity of CDK4/6 kinase can protect active Lgr5^+^/Olfm4^+^ intestinal stem cells and activate quiescent Bmi1^+^ stem cells, thereby promoting crypt regeneration *in vivo* and in organ culture models (69).

This is because: Firstly, CDK4/6 inhibition slows down the cell cycle, thereby increasing the opportunity for DNA repair and reducing the occurrence of mutations. Secondly, cell cycle-dependent kinases (CDKs) act as transcriptional co-regulators and can directly enhance the activity of pro-inflammatory transcription factors (such as NF-κB, STAT3, and AP-1), thereby promoting the expression of inflammatory factors. CDK inhibitor therapy may alleviate inflammation and promote epithelial tissue repair by disrupting this transcriptional activation loop dependent on CDKs (70).

The abnormal cell cycle regulatory system is a key factor leading to the unrestricted proliferation of cancer cells. The accumulation of mutations in intestinal epithelial stem cells results in the occurrence of cancer (71, 72). As mentioned earlier, in the context of IBD, the upregulation of cyclin D1 enables cells to grow rapidly even in the presence of limited proliferative signals, allowing cells to bypass critical cell cycle checkpoints and increasing the risk of cancer. In patients with gastrointestinal cancer, the cyclin D1 is over-activated. A study indicates that by promoting the K48-linked ubiquitination of cyclin D1 through MG53, it leads to its degradation by the proteasome, inducing G1 phase arrest, inhibiting the proliferation of cancer cells *in vitro*, and suppressing tumor growth in xenograft and AOM/DSS-induced colon cancer models (73). Similarly, in sporadic CRC with APC gene inactivation, cyclin D2 expression is rapidly upregulated following Apc loss. In APC-deficient mice, loss of cyclin D2 results in reduced intestinal epithelial cell proliferation, diminished crypt size, and suppression of tumor growth and development in Apc(Min/+) mice (74). However, the dysregulation of the CRC cell cycle system is not merely related to cyclin D and the G1 phase it regulates.

A large-scale single-cell sequencing analysis of 432,314 cells from 92 CRC patients and 24 IBD patients revealed through meta-program analysis that both malignant and non-malignant cells in CRC are enriched in the G2 to M phases of the cell cycle. This highlights that the abnormality of the cell cycle state is a key factor in the inflammatory-cancer transformation process (75).

The non-tumorous (with no histological dysplasia) but chronically inflamed colon epithelial cells of IBD patients. Large-scale whole-genome sequencing studies have provided conclusive evidence for this: the somatic mutation rate of the non-tumorous colon crypt epithelial cells of IBD patients is 2.4 times that of healthy controls (76). This sudden increase in mutational load may be due to continuous oxidative stress, ROS bursts, and inhibition of DNA repair mechanisms. When DNA damage (such as replication stress or double-strand breaks caused by reactive oxygen species) persists, cells will activate the G2/M checkpoint, blocking the cell cycle at the G2 phase for repair, to prevent the transmission of damaged DNA to daughter cells. TP53 alterations are a highly recurrent event in IBD-related cancers and occur in half of the dysplasia cases. TP53 mutations are already present in the dysplasia stage and are accompanied by chromosomal instability (60, 77).

Therefore, the G2/M phase enrichment observed in the non-tumor epithelial cells of IBD patients is essentially a stress- and protective cycle arrest response of the cells to cumulative DNA damage. Once the p53 function is lost, the G2/M checkpoint becomes ineffective. At this point, the inactivation of p53 and pRB can have a synergistic effect: the inactivation of pRB leads to errors in chromosome separation, while the inactivation of p53 allows these cells with already abnormal chromosome numbers to evade apoptosis and continue to proliferate (78).

Therefore, the G2/M phase enrichment observed in truly malignant CRC cells has undergone a fundamental change in nature: it is no longer a protective arrest but rather a brief transitional stage in the uncontrolled proliferation cycle. This phenomenon is universal across various cancers: based on integrated analyses of large-scale transcriptomic and proteomic data, it was found that in 9 types of human cancers (including colorectal cancer), the cyclin B and CDK1 genes have generally undergone transforming mutations/overexpression, while the core regulatory genes of the G2/M checkpoint (TP53, ATM, CDKN1A) have shown functional loss mutations, forming a reverse regulatory pattern of “checkpoint inhibition - cycle driving” (79).

In summary, in the transition from IBD to CRC, the “G2/M phase enrichment” phenotype carries fundamentally opposite biological meanings - the former represents the protective adaptation of the genome under inflammatory stress, while the latter represents the coexistence of cycle drive and genomic instability after checkpoint failure in a malignant steady state.

Further research has revealed that cyclin A2, along with its kinase partners CDK1 and CDK2, act as key initiators of DNA replication and mitosis, causing the cell cycle to transition from the S phase to the early M phase. In mice with mutant cell cycle protein A2, the decrease in the level of cell cycle protein A2 reduces the content of the RNA-binding domain at its C-terminus, which leads to the reduction of the MRE11 nuclease involved in meiotic recombination during the S phase. This damage results in defective unwinding of stalled replication forks, insufficient double-strand break repair, and errors in sister chromatid separation, thereby increasing tumor susceptibility (80). Additionally, in colitis-related cancer models, the absence of cell cycle protein A2 in intestinal epithelial cells exacerbates the formation of dysplasia and adenocarcinoma (81).

Meanwhile, these cell cycle protein kinase inhibitors, p21, p27 and p57, also play significant roles in cell cycle regulation.

P21 is regulated by p53 transcription and plays a central role in cell cycle arrest and DNA damage repair. When DNA damage occurs, the activation of p53 induces the expression of p21, thereby inhibiting CDK activity and promoting cell cycle arrest, providing conditions for DNA repair (82). In CRC, the expression of p21 is associated with tumor progression and favorable prognosis (83).

P27 mainly inhibits the cell cycle from entering the S phase by suppressing CDK2 and other factors. A clinical study has shown that high expression of p27 in the cytoplasm has a favorable prognosis for cancer patients. The loss of p27 may lead to uncontrollable proliferation and increased invasion, resulting in strong tumor invasiveness and poor prognosis (83, 84).

**Figure 2:**
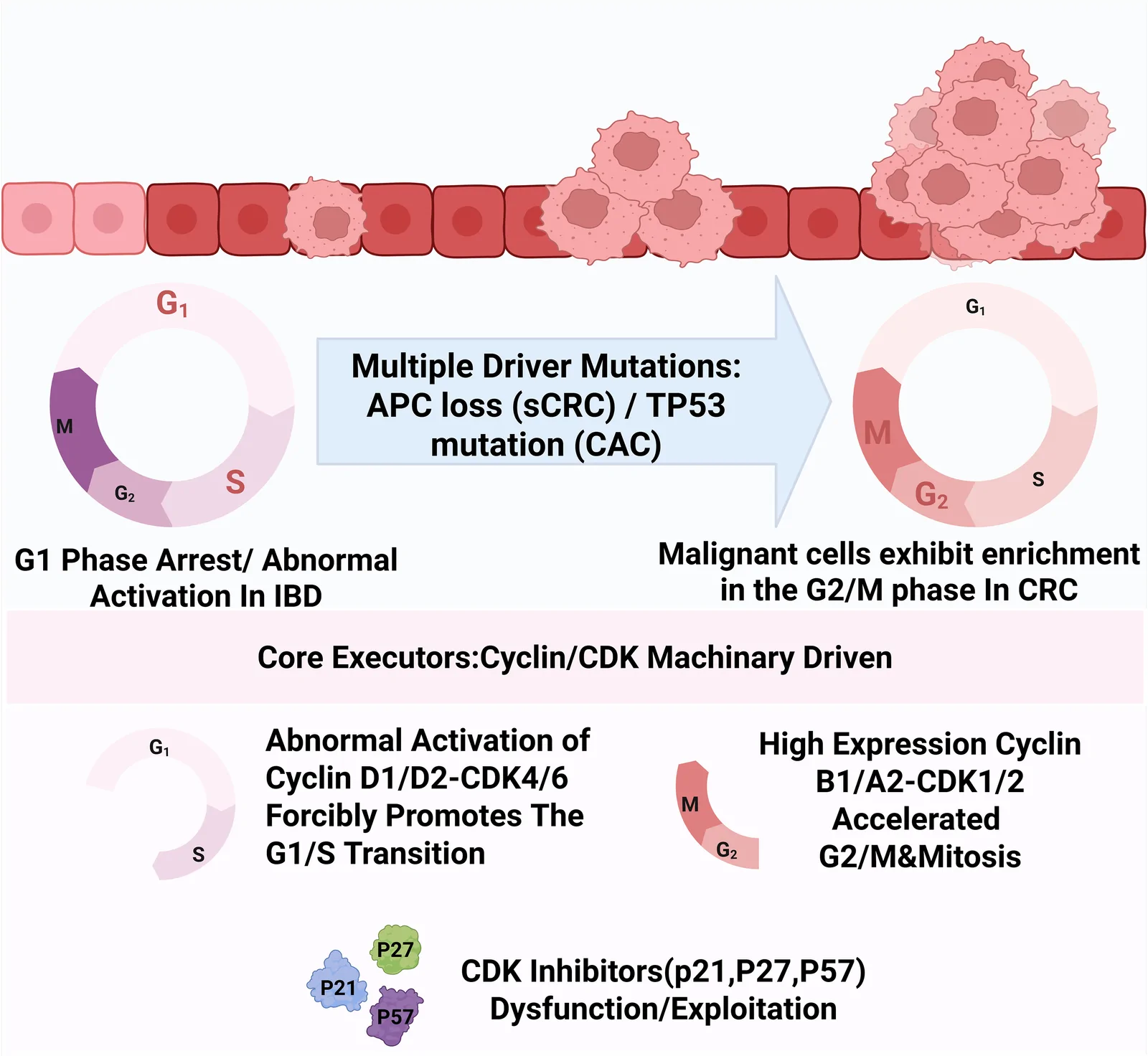
Cell cycle dysregulation in colorectal cancer leads to the occurrence of mutations. This schematic diagram shows that in the non-severe lesions of inflammatory bowel disease, there will be G1 phase arrest/abnormal activation, which leads to gene mutations (APC loss (sCRC)/TP53mutation (CAC)), thereby disrupting the normal balance of the cell cycle and causing an increase in the G2/M phase in colorectal cancer, promoting rapid cell proliferation. This abnormal development is driven by the core cell cycle executors: the abnormal activation of the cell cycle protein D1/D2 and the CDK4/6 complex enables the cells to transition from the G1 phase to the S phase. The high expression of the cell cycle protein B1/A2 - CDK1/2 complex accelerates the G2/M phase and the mitotic process. The abnormality of cell cycle inhibitors (such as p21, p27, and p57) is also an important component of cell cycle dysregulation.

Meanwhile, the CDK inhibitor p57 is also involved in various cellular processes, including transcription, apoptosis, differentiation, development, and migration, and affects the occurrence of CRC by controlling proliferation and promoting apoptosis (85). As a potent CDK inhibitor, p57 induces cell cycle arrest. In normal intestines, p57 is mainly expressed in quiescent reserve stem cells or cells at specific differentiation stages. In a study by Takeru Oka et al., a subgroup of slow-proliferating colorectal cancer stem cells (CSCs) with high Lgr5 expression specifically expressed p57. This specific expression of p57 in CSCs promotes regeneration after tumor chemotherapy, and this regeneration effect is relieved afterward. Therefore, we infer that p57^+^ CSCs are a highly malignant and adaptable subpopulation of CSCs during tumor evolution. They survive in treatment because of their slow proliferation characteristic and ultimately drive tumor recurrence and metastasis (86). Overall, these pieces of evidence indicate that the evolutionary process of cell cycle dysregulation in a chronic inflammatory environment is as shown in **Figure 2**, and this imbalance does not exist in isolation. It is composed of a series of core signaling pathways and activated by the inflammatory environment, tissue damage, mechanical damage, etc. In the following sections, we will systematically analyze the relevant key pathways, namely Rb–E2F, NF-κB, JAK–STAT, and Hippo–YAP. To clarify how they precisely and synergistically regulate the core cell cycle components and link the uncontrolled cell proliferation of tumor cells. It is important to clarify that the direct molecular links between ROS, DNA damage, and cell cycle checkpoint control (including G1/S and G2/M arrest) have been systematically discussed in Section 2. The following section (Section 3) shifts focus to four key signaling pathways—Rb-E2F, NF-κB, JAK-STAT, and Hippo-YAP—that serve as core downstream executors of the cell cycle machinery. While these pathways can be modulated by ROS in the inflammatory microenvironment, their regulation of cyclins, CDKs, and CKIs constitutes a more proximal and direct layer of control over cell cycle progression in CAC. Therefore, the detailed interplay between ROS and each pathway is not repeated here; instead, Section 3 concentrates on how these pathways, once activated, orchestrate the cell cycle dysregulation that drives the inflammation-cancer transition.

## 3 The relationship between different inflammation signaling pathways and the regulation of the cell cycle system

The transition from inflammation to cancer in colorectal carcinogenesis is a complex, multi-pathway process, at its core reliant on cell cycle dysregulation. Chronic inflammation acts as a critical driver, and the loss of normal cell cycle control promotes tumorigenesis through its direct impact on cellular proliferation and apoptotic resistance (87). Underlying this process, transcription factor networks are essential for generating the precise transcriptional programs that guide cell cycle progression.

### 3.1 Rb-E2F pathway

In the chronic inflammatory microenvironment of CAC, pro-inflammatory cytokines such as TNF-α and IL-6 activate NF-κB and STAT3 signaling (88, 89). Activation of these pathways has been shown to promote G1/S progression by transcriptionally upregulating key cell cycle regulators such as cyclin D1, thereby engaging the Rb-E2F axis (90, 91). The Rb-E2F pathway, as an important regulatory pathway of the cell cycle, functions during the transition from G1 to S phase. The upstream growth-promoting signals typically activate the cell cycle protein D-cyclin - CDK4/6 complex through the MAPK signaling pathway. These complexes phosphorylate and inhibit Rb. This releases the E2F factors, triggering the transcription of S-phase genes. The E2F itself enhances the activity of cell cycle protein E-cyclin - CDK2, ensuring the occurrence of S phase. In CRC, common pathological changes in this pathway include amplification of cyclin D1, overactive CDK4/6, or Rb, which leads to uncontrolled proliferation (92).

### 3.2 NF-κB pathway

NF-κB, as a classic inflammatory-related pathway, is activated by inflammatory factors (TNF-α, IL-1, LPS) (93). NF-κB promotes the development of CRC by regulating the expression of a series of target genes related to cell cycle progression (such as cyclin D1), angiogenesis (vascular endothelial growth factor, interleukin-8, cyclooxygenase-2), and invasion (matrix metalloproteinase-9) (94). Specifically, NF-κB enhances the transcription of cyclin D1 by directly binding to its gene promoter, thereby establishing a connection between inflammation and excessive proliferation (95).

### 3.3 JAK-STAT signaling pathway

The JAK-STAT pathway is critically involved in CAC, characterized by its persistent activation in response to inflammatory cytokines such as IL-6 (96). In CRC, sustained activation of STAT3 can promote tumor development, progression and metastasis (97). STAT3 enhances the transcription of Cyclin B1, and subsequently Cyclin B1 forms a complex with CDK1, thereby facilitating the transition to the G2/M phase to coordinate the process of entering mitosis, further reinforcing the role of the JAK-STAT signaling pathway in cell cycle dysregulation (98).

### 3.4 Hippo-YAP pathway

#### 3.4.1 YAP as a promoter of stemness and proliferation

Regarding the Hippo-YAP pathway, YAP functions as a transcriptional co-activator in the cell nucleus, exerting transcriptional regulatory effects. YAP is expressed in Lgr5^+^ basal columnar cells in intestinal crypts and regulates the expression of the intestinal stem cell marker Lgr5 through transcriptional mechanisms, expanding the progenitor and stem cell populations, adjusting the stem cell-like state and the regenerative capacity of reserve stem cells (99, 100). This function of YAP also has a crucial impact on CSCs. Through its transcriptional co-activating activity, YAP can enhance or inhibit the expression of genes related to CSCs. In a study by Xiao Qin et al., a single-cell analysis of 1107 colon organoids showed that YAP is crucial for the recovery of the revival colonic stem cells (revCSCs) (101). Unlike traditional proliferative CSCs, revCSCs prioritize cell survival rather than division, and play an important role in the occurrence, metastasis and non-genetic resistance of primary tumors. Under steady-state conditions, the inhibition of YAP mediated by the Hippo pathway is crucial for preventing abnormal crypt proliferation.

#### 3.4.2 Paradoxical tumor-suppressive activity of YAP

However, during tissue damage, the activation of YAP triggers the regeneration program, promoting the upregulation of the stem cell state and thereby facilitating the malignant transformation of tumors (102). Meanwhile, in colon tumors of the chemically-induced CAC model, the expression of YAP is significantly upregulated (103), and its activation promotes the proliferation of colon cancer cells and drives liver metastasis (104). However, the role of YAP in intestinal homeostasis and CRC remains controversial. Priscilla Zhang et al. challenged the traditional oncogenic view, demonstrating that the Hippo serine/threonine kinases LATS1/2 and MST1/2 (which can phosphorylate and inhibit YAP) are essential for maintaining the classical Wnt signaling pathway and normal stem cell activity. Their research found that inhibiting the Hippo pathway leads to a unique epithelial state characterized by weakened Wnt signaling, activation of the wound healing program, and increased expression of Klf6. Importantly, by deleting LATS1/2 or overexpressing YAP, tumor growth can be inhibited in organ cultures, xenograft models, and mouse CRC systems (105). The anti-tumor effect of YAP may be attributed to its promotion of the regenerative reprogramming of intestinal epithelial cells, rather than directly maintaining homeostasis. The anti-tumor effect of YAP may mainly stem from its ability to promote the regeneration and repair of intestinal epithelial cells. Studies have shown that in an inflammatory microenvironment, NF-κB p65 can inhibit the activity of YAP, while the presence of functional YAP is crucial for maintaining epithelial homeostasis and resisting inflammatory damage (106). Further research has discovered that YAP inhibits intestinal inflammation through epigenetic mechanisms: it can silence the expression of the histone H3K27me3 demethylase JMJD3, thereby inhibiting the activation of downstream pro-inflammatory genes, and thereby alleviating the pathological process of IBD (106). These findings collectively reveal that YAP, in an inflammatory context, exerts its protective and even anti-tumor effects by maintaining epithelial integrity and inhibiting excessive inflammatory responses.

#### 3.4.3 Reconciling the dual roles through cell cycle regulation

The cell cycle, as a key regulatory factor for cell growth and proliferation, provides a reasonable explanation for these seemingly contradictory research results. On one hand, the activation of YAP regulates the expression of multiple genes related to the cell cycle. After inflammation injury, YAP interacts with β-catenin, and the overexpression of YAP enhances the expression of β-catenin/Wnt response genes (such as Lgr5 and cyclin D1), indicating that inflammatory stimulation is associated with proliferation (107, 108). Meanwhile, YAP combines with YY1 and EZH2 to form a genomic complex, which inhibits CDK inhibitors such as p27, thereby promoting the occurrence of tumors (109).

On the other hand, one of the experiments mentioned in the above studies shows that inhibiting the Hippo pathway leads to weakened Wnt signaling, activation of the wound healing program, and increased expression of Krüppel-like factor 6(Klf6). The KLF6, as an important member of the Krüppel-like factor transcription factor family and a widely expressed gene, plays a key role in embryonic development, tissue repair, and tumor suppression. KLF6 exerts anti-tumor effects through dual mechanisms related to cell cycle regulation: first, it blocks the G1/S phase progression by binding and inhibiting the cyclin D1/CDK4 complex; second, it upregulates the CDK inhibitor p21 through a p53-independent pathway, thereby inducing G1 phase arrest and ultimately inhibiting tumor cell proliferation (110, 111).

This indicates that the hippo-YAP pathway in CRC does not merely promote or inhibit tumors. Its function depends on specific cellular environments, such as cell homeostasis, damage, specific gene mutations, and microenvironmental signals. This pathway can drive both the programs that promote regeneration and cancer proliferation, as well as the anti-cancer programs that inhibit proliferation and promote repair. The cell cycle acts as a bridge that links these two processes, clearly explaining this contradictory phenomenon.

### 3.5 Interactions and integration of signal networks

In CAC, the IL-6/TNF-α-mediated signaling network constitutes a crucial positive feedback loop. This loop, by simultaneously activating STAT3 and NF-κB, drives chronic inflammation and promotes uncontrolled cell cycle progression (112).

In CAC, the downstream effector molecule YAP of the Hippo pathway, which serves as the core mediator of inflammation and proliferation, is activated by inflammatory factors. Studies have shown that the IL-6/gp130 signal can directly phosphorylate YAP through Src family kinases and promote its nuclear translocation. This process is independent of the classical Hippo pathway (67).

After entering the nucleus, YAP directly interacts physically with NF-κB (p65), and YAP expands its functional scope as a co-activator. Studies have confirmed that in colorectal cancer cells, there is reciprocal activation between YAP and the NF-κB pathway. Both bind to the κB sites near the promoter regions of target genes, enhancing the recruitment efficiency of the transcriptional machinery and the degree of chromatin opening (113).

There is an interaction between STAT3 and YAP. In colitis and CAC models induced by DSS, the activation of YAP has been demonstrated to be an upstream event of STAT3 phosphorylation. YAP promotes the activation of STAT3 through some mechanism, thereby driving the proliferation of colonic epithelial cells (103).This relationship was further confirmed in normal human colon epithelial cells. YAP directly binds to STAT3 in the nucleus and regulates the expression of key genes (c-Myc and Cyclin D1) that control the G1/S phase, thereby promoting the proliferation of intestinal epithelial cells (114).

### 3.6 Wnt/β-catenin as the central integrator linking inflammation to cell cycle dysregulation

However, in the complex pathogenesis of CAC, the Wnt/β-catenin signaling pathway acts as the core coordinator. By forming a dense interaction network with the four key pathways, namely Rb–E2F, NF-κB, JAK–STAT, and Hippo–YAP, it jointly converts chronic inflammatory signals into persistent cell proliferation and anti-apoptotic phenotypes.

In the chronic inflammatory microenvironment, β-catenin accumulates abnormally in the cytoplasm and translocates into the nucleus to bind with TCF/LEF transcription factors, thereby activating a series of downstream target genes (115). Directly in the dysplastic tissues related to ulcerative colitis, it was observed that there was accumulation of β-catenin within the cells (a marker of Wnt activation), accompanied by upregulation of Cyclin D1 and c-myc expression, confirming that the Wnt pathway was activated at an early stage of carcinogenesis (116). The most crucial ones are c-Myc and Cyclin D1. These two target genes are precisely how the Wnt pathway triggers the cell cycle engine to drive the Rb–E2F signaling pathway.

The interaction between the Wnt pathway and the NF-κB pathway forms a direct bridge connecting inflammation and cancer. Activated NF-κB not only can upregulate various pro-inflammatory cytokines and anti-apoptotic genes, but also can directly promote the expression of Wnt ligands (such as WNT3A, Wnt5a) (117).

Meanwhile, the Wnt pathway can also indirectly stabilize the p65 subunit of NF-κB by inactivating GSK3β, thereby prolonging its transcriptional activity (118). More importantly, β-catenin is located upstream of NF-κB. β-catenin is necessary for the enhanced transcriptional activity of NF-κB and the nuclear localization of p65, and is closely related to the secretion of IL-8. It synergistically amplifies the pro-inflammatory and pro-cancer transcriptional program (119).

In the JAK-STAT pathway, chronic inflammation leads to elevated levels of cytokines such as IL-6, which continuously activate the JAK kinase and subsequently phosphorylate and activate STAT3. The activated STAT3 not only promotes cell survival and proliferation, but also interacts with the β-catenin/TCF4 complex, enhancing the transcriptional efficiency of Wnt target genes (120). Meanwhile, the gp130/JAK/STAT3 pathway regulates the expression of cell cycle inhibitory factors p16 and p21, thereby modulating the cell’s response to abnormal WNT signals (121).

This enables the inflammatory signals to be more effectively converted into proliferation outputs that are dependent on the Wnt pathway.

The Wnt pathway and the Hippo-YAP pathway have multiple cross-regulatory interactions (122). When Hippo is inactivated, YAP/TAZ enters the nucleus and binds to β-catenin, thereby synergistically activating proliferation-promoting genes such as AREG and BIRC5 (123, 124). Furthermore, the Wnt signal can affect the activity of YAP by regulating the stability of AMOTL2 (125, 126). This synergy is particularly crucial for maintaining the CAC tumor stem cells (127).

**Figure 3:**
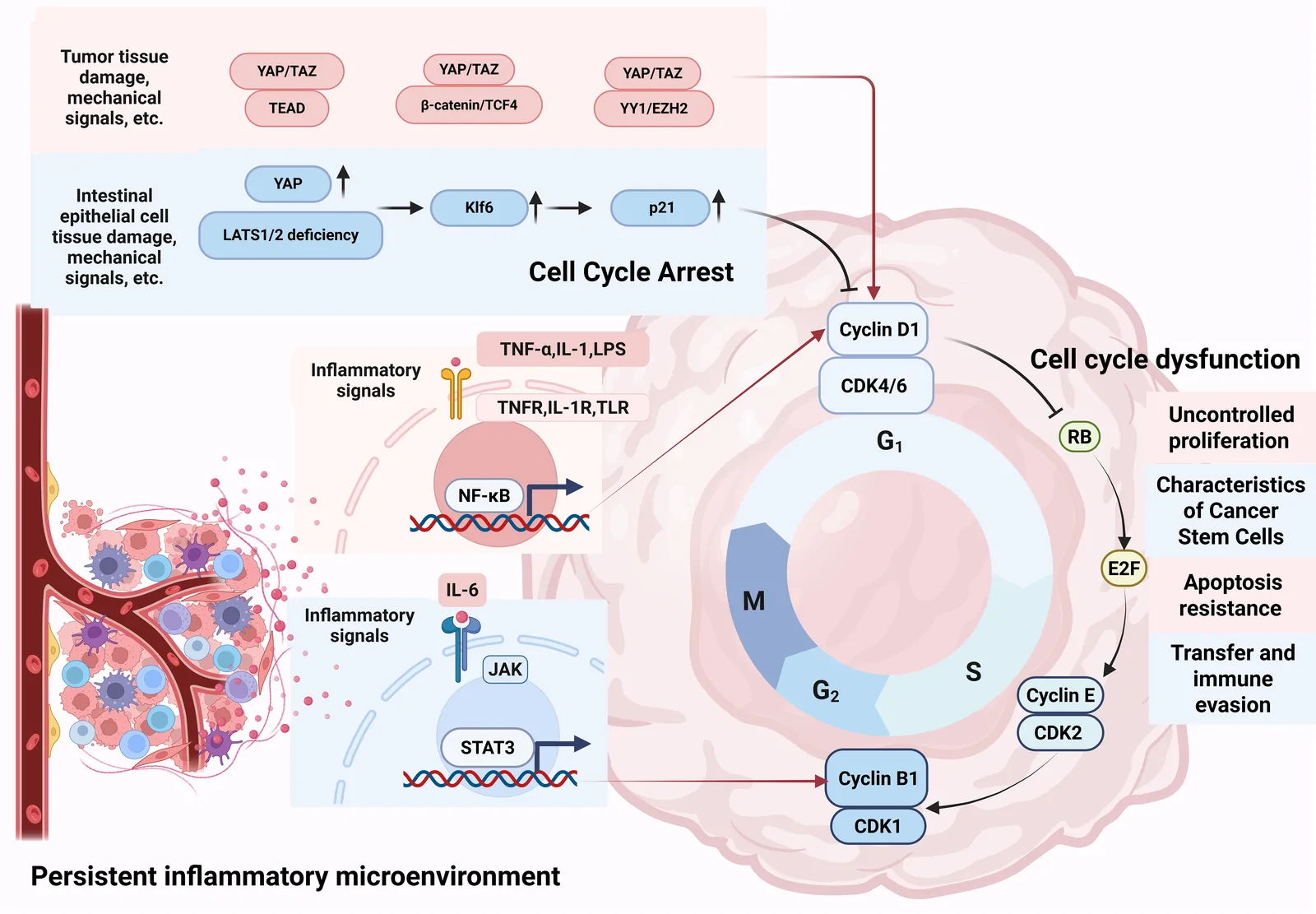
Chronic inflammatory microenvironment drives colorectal cancer through hijacking the core cell cycle engine. The NF-κB pathway directly responds to inflammatory signals and promotes Cyclin D1 transcription to drive the G1/S transition; the JAK-STAT pathway activates STAT3 through cytokine stimulation, upregulates Cyclin B1, and thereby promotes the G2/M phase progression; while the Hippo-YAP/TAZ pathway exhibits context-dependent dual roles: in the oncogenic state, it enhances Cyclin D1 expression, amplifies Wnt signaling, and inhibits CDK inhibitors to promote proliferation; however, in specific contexts, it inhibits Cyclin D1/CDK4 and induces cell cycle arrest through the KLF6/p21 axis. These signals are ultimately integrated by the Rb-E2F pathway, and the coordinated dysregulation of key nodes (such as Cyclin D1 amplification, over-activation of CDK4/6, and inactivation of Rb/p16) jointly leads to uncontrolled proliferation, tumor stem cell characteristics, resistance to apoptosis, and metastasis, etc., which are malignant phenotypes.

Although it has been confirmed that the intrinsic signaling pathways - Rb-E2F, NF-κB, JAK-STAT, and hippo-YAP/TAZ - play a crucial role in directly disrupting cell cycle control as shown in **Figure 3**, it must be recognized that their activities are profoundly regulated by external signals in the tumor microenvironment (TME). The following section will focus on the main cellular and molecular components of the colorectal cancer tumor microenvironment, explaining how they affect these core cell cycle mechanisms, thereby jointly coordinating tumor growth, immune evasion, and metastasis progression.

## 4 The main components of the tumor microenvironment in colorectal cancer and its effects on the cell cycle

In CRC, intricate crosstalk among various cellular components of the tumor microenvironment—including immune cells, fibroblasts, and endothelial cells—collectively regulates tumor cell growth, proliferation, and metastasis through diverse mechanism (128, 129).

There is a complex relationship between cyclins/CDK and the tumor microenvironment. The components of the tumor microenvironment can regulate the cell cycle system, thereby reshaping the tumor microenvironment and promoting tumor growth (130–132). For example: The immune components in the tumor microenvironment, including T cells, B cells, and macrophages, directly or indirectly affect the cell cycle of tumor cells by secreting soluble factors such as cytokines and chemokines (128, 133–135).

At the same time, the cell cycle status of tumor cells also affects the immune response (136). Taking T cells as an example: Although T cells play a key role in the tumor immune microenvironment, they recognize tumor antigens and mediate immune surveillance. However, in a study by Ona-Maria Thomas et al., the low expression of p21 in CD4^+^ T cells infiltrating CRC patients was associated with a decreased cancer-specific survival rate. Similarly, in a mouse CRC model, the Th1 cells lacking p21 exhibited a depleted phenotype, characterized by the accumulation of effector/effector memory T cells and the loss of CD27/CD28 expression. The lack of p21 in type 1 helper T cells promoted tumor growth in the mouse CRC model. P21 mainly acts to inhibit CDK4/6, and after replacing P21 with palbociclib (a CDK4/6 inhibitor) treatment, over-transplanting CD4^+^ T cells lacking p21 into Rag1^-^/^-^ mice models restored cytotoxic function and prevented the exhaustion phenomenon (137). Other cyclin kinases also affect the infiltration of other immune cells in the tumor. For example, the study conducted by Li Ping Guan et al. showed that the high expression of CDK9, CDK14, and CDK17 was positively correlated with CD8^+^ T cell infiltration, while CDK1, CDK4, and CDK8 were associated with reduced Treg infiltration (130).

It is worth noting that among the aforementioned kinases positively correlated with CD8^+^ T cell infiltration, CDK9 has been studied the most thoroughly, and its function exhibits a bidirectional and crucial characteristic. On one hand, in the context of IBD, systemic inhibition of CDK9 can significantly improve the histological damage of immune-mediated colitis, and the mechanism is related to the targeted inhibition of IFN-γ and IL-17A derived from colonic CD4^+^ T cells, suggesting that CDK9 is involved in driving intestinal inflammatory responses (138). On the other hand, in established colorectal cancer, high expression of CDK9 is closely related to immune escape: in microsatellite stable (MSS) metastatic colorectal cancer, the expression level of CDK9 is negatively correlated with the infiltration of CD8^+^ T cells, while positively correlated with the exhaustion of CD8^+^ T cells (139).

## 5 Targeted strategies for the “inflammation-cancer” transformation of colorectal cancer cell cycle regulation

During the transformation from inflammation to cancer in CRC, the dysregulation of the cell cycle plays a central role. Its abnormal activation not only promotes the malignant proliferation of epithelial cells but also advances the “inflammation-cancer” process by reshaping the tumor immune microenvironment and regulating chronic inflammatory signaling pathways. As a common feature of cancer, the dysregulation of the cell cycle in CRC is manifested as elevated levels of cell cycle-related proteins/CDKs or reduced activity of endogenous inhibitors, leading to uncontrolled cell proliferation. CDK inhibitors effectively inhibit the proliferation of abnormal cancer cells by targeting and blocking the activity of CDK kinases. Moreover, CDKs are involved in promoting the expression of pro-inflammatory genes, thereby creating a microenvironment that supports tumor growth. Therefore, therapeutic strategies targeting CDKs provide new approaches for the treatment of CRC, including anti-proliferation and immune regulation (70, 140).

The three major FDA-approved CDK4/6 inhibitors are: Palbociclib (Brand name: Ibrance) - Used for metastatic breast cancer, usually in combination with hormonal therapy. Ribociclib (Brand name: Kisqali) - Used for both early and metastatic breast cancer alongside hormonal therapy. Abemaciclib (Brand name: Verzenio) - Used for both early and metastatic breast cancer; can be used in combination with hormonal therapy or as a standalone treatment (141). At the same time, they have shown great potential in the treatment of CRC. For instance, Wei Xiaoli Wen et al. reported that abemaciclib inhibits the growth of CRC by degrading the YAP1 protein and weakening the cancer stem cell properties and chemotherapy resistance (142). Michael S. Lee et al. discovered that the combination of palbociclib with a MEK inhibitor showed a synergistic effect in KRAS mutant CRC models, functioning by inhibiting the ERK signaling pathway, down-regulating the expression of cyclin D, and enhancing G1 phase arrest (143). Similarly, in a collaborative clinical study targeting RAS mutant metastatic colorectal cancer (accounting for approximately 45% of cases), the combination regimen of the MEK1/2 inhibitor Binimetinib and the CDK4/6 inhibitor Palbociclib was evaluated in 18 patient-derived xenograft models and a safety import cohort consisting of 6 patients. This combination regimen induced tumor regression in 60% of the patient-derived xenograft models, highlighting its significant clinical translational potential (144). Additionally, Razia Aslam et al. demonstrated that Ribociclib combined with the PI3K inhibitor Alpelisib exhibits synergistic anti-proliferative effects in CRC cell lines, particularly in those with PIK3CA/KRAS mutations (145).

In addition to directly causing G1 phase arrest, it is also worth noting that the disrupted cell cycle proteins can alter the infiltration and function of immune cells, thereby creating an environment conducive to tumor escape (146). More and more evidence indicates that the degree of T cell infiltration is associated with the tumor cell cycle. As cyclins are pivotal drivers of both proliferation and immune regulation, targeting them represents a promising strategy to enhance immunotherapy in CRC. Specifically, inhibiting the cell cycle, particularly with CDK4/6 inhibitors, can remodel the tumor microenvironment and increase sensitivity to immune checkpoint blockade (136).

In colorectal cancer, patients with low PD-L1 expression have significantly lower objective response rates to anti-PD-1 monotherapy, and even show no response at all, indicating that the level of PD-L1 expression is positively correlated with the efficacy of immunotherapy (147). This phenomenon stems from the physiological function of the PD-1/PD-L1 pathway: After PD-L1 binds to PD-1, it inhibits T-cell signaling by recruiting the SHP-2 phosphatase, thereby maintaining immune tolerance (148).

The expression level of PD-L1 largely reflects the presence of an active immune response in the tumor microenvironment, particularly the activity of the IFNγ signaling pathway. In an effective anti-tumor immune response, activated CD8+ T cells secrete large amounts of IFNγ. After binding to its receptor, IFNγ activates downstream transcription factors IRF1 and STAT1 through the JAK-STAT signaling pathway, thereby inducing the upregulation of PD-L1 expression in tumor cells and myeloid cells (149, 150). This upregulation of PD-L1 induced by the immune response is referred to as “adaptive immune resistance” (151). Therefore, high PD-L1 expression is usually regarded as an indirect indicator, suggesting the presence of active T-cell infiltration within the tumor and ongoing immune attack; PD-L1 is closely related to the immune microenvironment rich in CD8^+^ T cells, Th1 cytokines, and interferon. Conversely, low PD-L1 expression often indicates the absence of T-cell infiltration in the tumor microenvironment, or a defect in the IFNγ signaling pathway, resulting in the inability to effectively induce PD-L1 expression even when there are a few T cells present. In such cases, anti-PD-1 monotherapy fails due to the lack of an effective target and effector cells (152–154).

It is worth noting that the natural high PD-L1 level reflects the adaptive resistance of the tumor to immune attack, while the upregulation of PD-L1 induced by CDK4/6 inhibitors is an actively created window for immunotherapy.

Based on this, palbociclib inhibits the kinase activity of CDK4/6, hinders the phosphorylation of SPOP protein mediated by cyclin D-CDK4, thereby promoting the degradation of SPOP through the APC/C^Cdh1^ complex, resulting in a reduction in the ubiquitination degradation of PD-L1, and subsequently increasing the level of PD-L1 protein on the tumor cell surface; the anti-PD-1 antibody, with its extremely high affinity for PD-1, efficiently occupies the PD-1 on the surface of T cells, even if the tumor cells highly express PD-L1 due to the action of CDK4/6 inhibitors, these PD-L1 molecules cannot bind to the PD-1 that has been occupied by the antibody, thus unable to exert the immunosuppressive function, leading to the complete disintegration of the tumor’s immune escape mechanism. This process explains why an increase in PD-L1 level, in the presence of anti-PD-1 antibodies, can actually enhance the therapeutic effect. This strategy achieved complete tumor remission and a significant increase in survival rate in the CT26 mouse model (155).

In another study, it was also pointed out that after using CDK4/6 inhibitors, the expression of MHC I class molecules and tumor-associated antigens on tumor cells was upregulated, thereby enhancing the recognition ability of CD8^+^ T cells for tumor cells; DNA damage accumulation was induced, the cGAS-STING signaling pathway was activated, and type I interferons were promoted. IFN-I, as a key immune stimulatory molecule, can promote the maturation and antigen-presenting ability of dendritic cells, and promote the activation and infiltration of CD8^+^ T cells. At the same time, these drugs can also inhibit the expansion of regulatory T cells and promote the maturation and activation of DCs, thereby further enhancing the initiation and recruitment of T cells (156).These immunostimulatory effects help break the immunosuppressive balance established by chronic inflammation, thereby converting an immunologically “cold” tumor into a more immunoreactive “hot” tumor.

Apart from CDK4/6, other members of the CDK family have also gradually become new focuses in the research on colorectal cancer.

**Figure 4:**
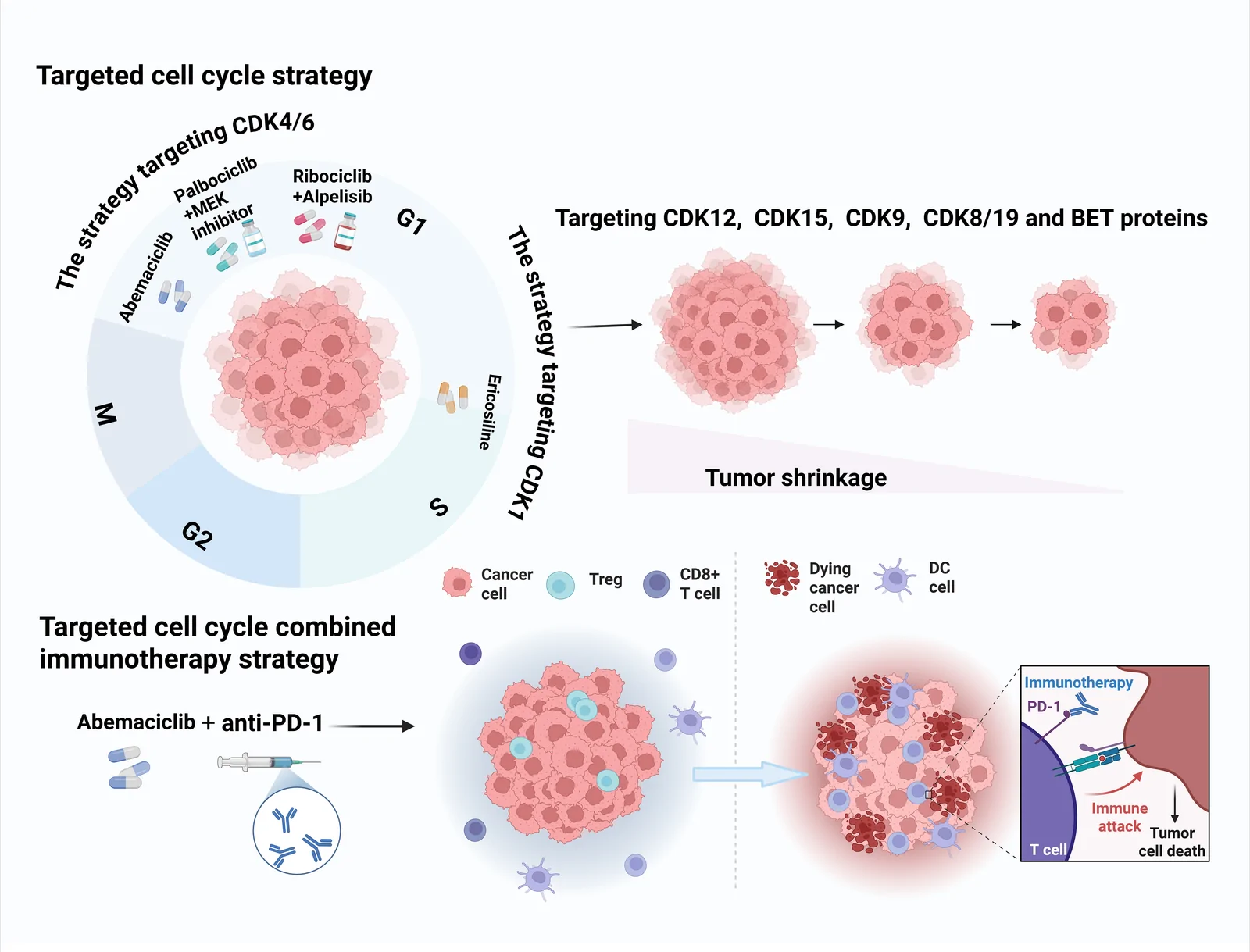
Targeted treatment strategies for colorectal cancer that target the cell cycle. This illustration explains the mechanism of action of targeted cell cycle therapy and the combined use of targeted cell cycle therapy and immunotherapy. CDK4/6 inhibitors directly induce tumor cell cycle arrest and death. The combination of CDK4/6 inhibitors (such as Abemaciclib) with anti-PD-1 immunotherapy can further enhance tumor antigen presentation, activate dendritic cells, and increase the killing activity of CD8^+^ T cells. Additionally, there are strategies that target other CDK members (CDK12/15/9), CDK8/19, and BET proteins in combination, which can also inhibit tumor growth.

CDK1 is highly expressed in CRC, and its levels correlate with immune infiltration and clinical outcomes. It is involved in cell cycle and p53-related pathways, and its inhibition suppresses the proliferation, migration, and invasion of CRC cells *in vitro*. In a translational screening experiment, Ericosiline was identified as a direct CDK1 inhibitor capable of inhibiting the growth and invasive capacity of HCT116 cells (157). CDK12 regulates CSCs self-renewal in various cancers. Targeting CDK12 can reverse the malignant phenotype of CRC by inhibiting the Wnt/β-catenin signaling pathway, providing a new strategy, particularly for targeting CSCs considered the root of tumorigenesis, growth, and metastasis (158). CDK15 promotes the progression of CRC by phosphorylating PAK4 and regulating the β-catenin/MEK-ERK signaling pathway (159). The CDK9 inhibitor CDKI-73 induces caspase−independent apoptosis in HCT116 and HT29 cells, significantly inhibiting tumor growth in the HCT116 tumor transplantation model (160). In the clinical setting, the selective CDK9 inhibitor KB-0742 has shown on−mechanism single−agent anti−tumor activity and a manageable safety profile in heavily pre−treated patients, including those with colorectal cancer, in an ongoing Phase 1/2 trial (161). These findings collectively support the translational potential of CDK9 inhibition as a therapeutic strategy for colorectal cancer. Moreover, CDK8/19 inhibitors not only specifically target and regulate oncogene−associated gene expression in CRC (162)but also show a synergistic growth inhibitory effect when combined with BET proteins in CRC models (163). **Figure 4** presents the overall situation of this targeted cell cycle or combined targeted cell cycle and immunotherapy strategy for colon cancer, and also shows some new research related to the CDK family that can lead to tumor shrinkage at the CDK target sites.

## 6 Emerging mechanisms linking inflammation to disruption of the cell cycle

### 6.1 cGAS-STING pathway

Errors during the cell cycle process are the main endogenous source that activates cGAS-STING (164, 165). The abnormal double-stranded DNA in the cytoplasm acts as a danger signal, activating the cGAS-STING signaling pathway. After activation, it recruits the TBK1 kinase, phosphorylates the transcription factors IRF3 and NF-κB, and ultimately induces the production of IFN-I and various pro-inflammatory cytokines (such as TNF-α, IL-6) (166, 167). During the process of colitis-cancer transformation, that is, in the early and middle stages of tumor formation, the weakening of the cGAS-STING pathway is associated with better survival rates and more immune infiltration in cancer patients, suggesting that its activation may have a carcinogenic effect (168). However, the cGAS-STING pathway is not merely a carcinogenic factor. In the later stage of tumor formation, dendritic cells, upon sensing dsDNA from tumor cells through the cGAS-STING pathway, can mature and efficiently cross-present tumor antigens, thereby activating CD8^+^ T cells and mediating the immune clearance of the tumor (169).

### 6.2 Ferroptosis

There is a precise bidirectional regulatory relationship between ferroptosis and the cell cycle. On one hand, the state of cell cycle arrest can significantly alter the sensitivity of cells to ferroptosis. Studies have confirmed that by stabilizing p53 or inducing G1 phase arrest using CDK4/6 inhibitors (such as Palbociclib), cancer cells can become highly sensitive to inhibitors that directly target GPX4 (170).

On the other hand, some studies have presented contradictory viewpoints, suggesting that the induction of cell cycle arrest inhibits the formation of lipid droplets through diacylglycerol acyltransferase, in order to prevent the accumulation of polyunsaturated fatty acids in the arrested cells, thereby inhibiting ferroptosis (171). This difference is likely to stem from the specific mechanisms of stagnation induction, the types of cells, and the background of the microenvironment. However, it is precisely this regulation of the sensitivity to ferroptosis by the cell cycle state that has opened up new possibilities for the current situation of chemotherapy resistance in tumor cells.

### 6.3 Gut microbiota and their metabolites

In the process of CAC, the gut microbiome undergoes significant dysregulation. Among the pathogenic bacteria, Escherichia coli carrying the polyketide synthase gene island (pks+) can specifically alkylate the adenine bases on the double-stranded DNA of host cells, thereby causing severe DNA damage such as double-strand breaks (172, 173). Apart from the direct genotoxic effects, the metabolites produced by the gut microbiota constitute another important carcinogenic pathway. In patients with CAC, the production of beneficial metabolites such as short-chain fatty acids (SCFAs, especially butyrate) is reduced. They help maintain a healthy intestinal environment by inhibiting histone deacetylases and are also immunomodulators (174). However, some studies have indicated that its effect may not be completely protective in cases of colorectal cancer (175, 176).

### 6.4 Telomere dysfunction and replication stress

In IBD, the intestinal epithelium undergoes repeated damage, forcing the ISCs located at the bottom of the crypts to divide at a frequency far exceeding the physiological level in order to compensate for the lost epithelial cells and maintain the barrier integrity.

This accelerated stem cell renewal is a necessary compensatory mechanism for the body to respond to damage, but the cost is extremely high (177).

Each cell division is accompanied by an end-replication problem, that is, because the DNA polymerase cannot completely replicate the ends of the linear chromosomes, the telomere DNA sequence undergoes progressive shortening (178). Under the CAC condition, the excessive proliferation of intestinal stem cells directly leads to the accelerated depletion of telomeres (179). The dysfunctional telomeres are not only unstable on their own, but also may fuse with other chromosome ends, resulting in extensive chromosomal deletions, amplifications and rearrangements (180).

### 6.5 Long non-coding RNAs and circular RNAs

In the context of CAC and the broader CRC, multiple oncogenic lncRNAs have been demonstrated to be capable of overcoming the critical limitations on G1/S phase transition. For instance, oncogenic lncRNAs such as CCAT1, CRNDE, and PVT1 are closely associated with tumor progression and the regulation of the cell cycle. CCAT1 has a functional association with miR-145 in colon cancer, promoting the proliferation and invasion of tumor cells (181, 182). CRNDE acts as a ceRNA sponge to bind to miR-136, releasing its inhibition on the transcription factor E2F1, thereby indirectly promoting the expression of cell cycle-related genes (183). PVT1 can directly positively regulate the expression of cyclin D1 and CDK4, and functions as a ceRNA to exert its oncogenic effect (184). Furthermore, between lncRNA MEF and the oncogene c-Myc, c-Myc can transcriptionally activate MEF, while MEF can in turn enhance the expression and stability of c-Myc, jointly driving the transcription of various cell cycle genes including Cyclin D, and accelerating tumor formation (185). On the contrary, tumor suppressor lncRNAs such as MEG3 exert the opposite effect. By down-regulating the expression of Cyclin D1, they effectively arrest the cell cycle at the G1 phase and inhibit tumor growth (186, 187).

In the CAC model, the circRNA derived from PRKAR2A activates the classical Wnt signaling pathway, thereby promoting the malignant transformation of colitis to colorectal cancer (188). It is worth noting that circRNA can also precisely regulate the cell cycle process by directly binding to CDK proteins. For instance, circHIPK3 in CRC can directly bind and stabilize the CDK1 protein, thereby inhibiting its degradation. On the contrary, some circRNAs inhibit tumor growth by upregulating CDK inhibitory proteins such as p27. For example, circRNA_0025202 can upregulate p27 to inhibit the activity of CDK2, achieving precise negative regulation of the cell cycle (189).

## 7 Discussion

Our comprehensive analysis indicates that the cell cycle disorder in CRC goes far beyond uncontrolled proliferation. It acts as a medium to integrate inflammatory signals, genomic instability, stem cell properties, and immune evasion into the carcinogenic process. The Rb-E2F, NF-κB, JAK-STAT, and Hippo-YAP pathways play a crucial role in key cell cycle proteins and their dependent kinases and kinase inhibitors. Particularly, cyclin D1, A2, B1, and their CDK complexes. This makes chronic inflammation not only directly promote proliferation signals but also cause checkpoint control failure. Studies have found that malignant cells in CRC are mainly concentrated in the G2/M phase, while the epithelial cells of IBD have abnormal G1 phase, indicating that IBD patients have already experienced cell cycle dysregulation in precancerous changes. The dual functions of CDK inhibitors (p21, p27, p57) in intestinal homeostasis and carcinogenesis process indicate the complexity of targeting cell cycle regulation in treatment. Although these proteins usually maintain the integrity of epithelial cells, cell cycle dysregulation may paradoxically promote tumor progression. The p21 deficiency in CD4^+^ T cells promotes cell exhaustion and tumor growth, and can be reversed by CDK4/6 inhibition, highlighting the importance of considering the immune cell cycle status in treatment design.

Our review also integrates inflammatory-cancer intervention strategies targeting the cell cycle system as the plan: Abemaciclib is used for tumors triggered by YAP1, palbociclib combination is used for KRAS mutant CRC, and ribociclib combined with PI3K inhibitors is used for PIK3CA mutant cases. Besides CDK4/6, new targets include CDK1, CDK12, and CDK8/19.

CDK4/6 inhibitors can overcome the resistance mechanism resulting from immune rejection through the strategic combination of immune checkpoint inhibitors, especially in microsatellite stable CRC, where the benefits of immunotherapy are still limited.

Intervening in cyclin/CDK complexes in the bodies of high-risk IBD patients may delay or even prevent the occurrence of carcinogenesis. Targeting specific subgroups of colon CSCs to adjust the cell cycle (such as p57^+^ cells with slow proliferation) and targeting YAP-mediated anti-tumor effects may disrupt the reprogramming process that enables tumors to adapt to the environment.

In conclusion, targeting cell cycle regulation in CRC is a promising approach that can address the fundamental drivers of the inflammatory-cancer transition. By strategically regulating specific CDK activity in the context of immunity and inflammation, it may break the vicious cycle that promotes the occurrence of CAC.

## References

[1] Bray F Laversanne M Sung H Ferlay J Siegel RL Soerjomataram I . Global cancer statistics 2022: Globocan estimates of incidence and mortality worldwide for 36 cancers in 185 countries. CA Cancer J Clin. (2024) 74:229–63. doi: 10.3322/caac.21834 38572751

[2] Siegel RL Wagle NS Star J Kratzer TB Smith RA Jemal A . Colorectal cancer statistics, 2026. CA Cancer J Clin. (2026) 76:e70067. doi: 10.3322/caac.70067 41769777 PMC12951547

[3] Eng C Yoshino T Ruíz-García E Mostafa N Cann CG O'Brian B . Colorectal cancer. Lancet. (2024) 404:294–310. doi: 10.1016/s0140-6736(24)00360-x 38909621

[4] Feng Y Jin H Guo K Wasan HS Ruan S Chen C . Causes of death after colorectal cancer diagnosis: A population-based study. Front Oncol. (2021) 11:647179. doi: 10.3389/fonc.2021.647179 33859947 PMC8042257

[5] Li Q Geng S Luo H Wang W Mo YQ Luo Q . Signaling pathways involved in colorectal cancer: Pathogenesis and targeted therapy. Signal Transduct Tgt Ther. (2024) 9:266. doi: 10.1038/s41392-024-01953-7 39370455 PMC11456611

[6] Cao JH Cao CH Lin JL Li SY He LJ Han K . Neil1 drives the initiation of colorectal cancer through transcriptional regulation of Col17a1. Cell Rep. (2024) 43:113654. doi: 10.1016/j.celrep.2023.113654 38175757

[7] Acevedo-León D Monzó-Beltrán L Pérez-Sánchez L Naranjo-Morillo E Gómez-Abril S Estañ-Capell N . Oxidative stress and DNA damage markers in colorectal cancer. Int J Mol Sci. (2022) 23. doi: 10.3390/ijms231911664 36232966 PMC9569897

[8] Schmitt M Greten FR . The inflammatory pathogenesis of colorectal cancer. Nat Rev Immunol. (2021) 21:653–67. doi: 10.1038/s41577-021-00534-x 33911231

[9] Matas J Kohrn B Fredrickson J Carter K Yu M Wang T . Colorectal cancer is associated with the presence of cancer driver mutations in normal colon. Cancer Res. (2022) 82:1492–502. doi: 10.1158/0008-5472.Can-21-3607 35425963 PMC9022358

[10] Lieu CH Golemis EA Serebriiskii IG Newberg J Hemmerich A Connelly C . Comprehensive genomic landscapes in early and later onset colorectal cancer. Clin Cancer Res. (2019) 25:5852–8. doi: 10.1158/1078-0432.Ccr-19-0899 31243121 PMC6774873

[11] Veettil SK Wong TY Loo YS Playdon MC Lai NM Giovannucci EL . Role of diet in colorectal cancer incidence: Umbrella review of meta-analyses of prospective observational studies. JAMA Netw Open. (2021) 4:e2037341. doi: 10.1001/jamanetworkopen.2020.37341 33591366 PMC7887658

[12] Carr PR Weigl K Jansen L Walter V Erben V Chang-Claude J . Healthy lifestyle factors associated with lower risk of colorectal cancer irrespective of genetic risk. Gastroenterology. (2018) 155:1805–15:e5. doi: 10.1053/j.gastro.2018.08.044 30201362 PMC6279591

[13] Rajamäki K Taira A Katainen R Välimäki N Kuosmanen A Plaketti RM . Genetic and epigenetic characteristics of inflammatory bowel disease-associated colorectal cancer. Gastroenterology. (2021) 161:592–607. doi: 10.1053/j.gastro.2021.04.042 33930428

[14] Chen J Terry MB Dalerba P Hur C Hu J Yang W . Environmental drivers of the rising incidence of early-onset colorectal cancer in the United States. Int J Cancer. (2024) 154:1930–9. doi: 10.1002/ijc.34887 38339887 PMC10984757

[15] Gausman V Dornblaser D Anand S Hayes RB O'Connell K Du M . Risk factors associated with early-onset colorectal cancer. Clin Gastroenterol Hepatol. (2020) 18:2752–9:e2. doi: 10.1016/j.cgh.2019.10.009 31622737 PMC7153971

[16] Nagao-Kitamoto H Kitamoto S Kamada N . Inflammatory bowel disease and carcinogenesis. Cancer Metastasis Rev. (2022) 41:301–16. doi: 10.1007/s10555-022-10028-4 35416564

[17] Hisamatsu T Miyoshi J Oguri N Morikubo H Saito D Hayashi A . Inflammation-associated carcinogenesis in inflammatory bowel disease: Clinical features and molecular mechanisms. Cells. (2025) 14. doi: 10.3390/cells14080567 40277893 PMC12025475

[18] Jess T Simonsen J Jørgensen KT Pedersen BV Nielsen NM Frisch M . Decreasing risk of colorectal cancer in patients with inflammatory bowel disease over 30 years. Gastroenterology. (2012) 143:375–81:e1. doi: 10.1053/j.gastro.2012.04.016 22522090

[19] Lutgens MW van Oijen MG van der Heijden GJ Vleggaar FP Siersema PD Oldenburg B . Declining risk of colorectal cancer in inflammatory bowel disease: An updated meta-analysis of population-based cohort studies. Inflammation Bowel Dis. (2013) 19:789–99. doi: 10.1097/MIB.0b013e31828029c0 23448792

[20] Shah SC Itzkowitz SH . Colorectal cancer in inflammatory bowel disease: Mechanisms and management. Gastroenterology. (2022) 162:715–30:e3. doi: 10.1053/j.gastro.2021.10.035 34757143 PMC9003896

[21] Dan WY Zhou GZ Peng LH Pan F . Update and latest advances in mechanisms and management of colitis-associated colorectal cancer. World J Gastrointest Oncol. (2023) 15:1317–31. doi: 10.4251/wjgo.v15.i8.1317 37663937 PMC10473934

[22] Nadeem MS Kumar V Al-Abbasi FA Kamal MA Anwar F . Risk of colorectal cancer in inflammatory bowel diseases. Semin Cancer Biol. (2020) 64:51–60. doi: 10.1016/j.semcancer.2019.05.001 31112753

[23] Fanizza J Bencardino S Allocca M Furfaro F Zilli A Parigi TL . Inflammatory bowel disease and colorectal cancer. Cancers Bsl. (2024) 16. doi: 10.3390/cancers16172943 39272800 PMC11394070

[24] Kapadia A Joshi D Chavda A Bhatt P . From inflammation to carcinogenesis: Distinct pathways and clinical implications of ibd-associated colorectal cancer compared with sporadic crc. Pathol Res Pract. (2025) 275:156249. doi: 10.1016/j.prp.2025.156249 41043201

[25] Lopetuso LR Murgiano M Mantuano E Schiavone V Costa A Mascianà G . The molecular landscape of inflammation in inflammatory bowel disease (ibd): Targets for precision medicine. Biomedicines. (2025) 13. doi: 10.3390/biomedicines13112738 41301831 PMC12650290

[26] Wéra O Lancellotti P Oury C . The dual role of neutrophils in inflammatory bowel diseases. J Clin Med. (2016) 5. doi: 10.3390/jcm5120118 27999328 PMC5184791

[27] Lin Y Cheng L Liu Y Wang Y Wang Q Wang HL . Intestinal epithelium-derived Batf3 promotes colitis-associated colon cancer through facilitating Cxcl5-mediated neutrophils recruitment. Mucosal Immunol. (2021) 14:187–98. doi: 10.1038/s41385-020-0297-3 32467604

[28] Carnevale S Ponzetta A Rigatelli A Carriero R Puccio S Supino D . Neutrophils mediate protection against colitis and carcinogenesis by controlling bacterial invasion and Il22 production by γδ T cells. Cancer Immunol Res. (2024) 12:413–26. doi: 10.1158/2326-6066.Cir-23-0295 38349973 PMC10985471

[29] De Cicco P Sanders T Cirino G Maloy KJ Ianaro A . Hydrogen sulfide reduces myeloid-derived suppressor cell-mediated inflammatory response in a model of Helicobacter hepaticus-induced colitis. Front Immunol. (2018) 9:499. doi: 10.3389/fimmu.2018.00499 29636751 PMC5880908

[30] Lucafò M Curci D Franzin M Decorti G Stocco G . Inflammatory bowel disease and risk of colorectal cancer: An overview from pathophysiology to pharmacological prevention. Front Pharmacol. (2021) 12:772101. doi: 10.3389/fphar.2021.772101 34744751 PMC8563785

[31] Triantaphyllopoulos KA Ragia ND Panagiotopoulou ME Sourlingas TG . Integrating inflammatory and epigenetic signatures in ibd-associated colorectal carcinogenesis: Models, mechanisms, and clinical implications. Int J Mol Sci. (2025) 26. doi: 10.3390/ijms26199498 41096771 PMC12524939

[32] Sakurai N Shibata T Nakamura M Takano H Hayashi T Ota M . Influence of Mif polymorphisms on Cpg island hyper-methylation of Cdkn2a in the patients with ulcerative colitis. BMC Med Genet. (2020) 21:201. doi: 10.1186/s12881-020-01140-9 33046033 PMC7552536

[33] Emmett RA Davidson KL Gould NJ Arasaradnam RP . DNA methylation patterns in ulcerative colitis-associated cancer: A systematic review. Epigenomics. (2017) 9:1029–42. doi: 10.2217/epi-2017-0025 28621161

[34] Knudsen ES Witkiewicz AK Rubin SM . Cancer takes many paths through G1/S. Trends Cell Biol. (2024) 34:636–45. doi: 10.1016/j.tcb.2023.10.007 37953123 PMC11082069

[35] Fernandes MS Carneiro F Oliveira C Seruca R . Colorectal cancer and Rassf family--a special emphasis on Rassf1a. Int J Cancer. (2013) 132:251–8. doi: 10.1002/ijc.27696 22733432

[36] Riffet M Eid Y Faisant M Fohlen A Menahem B Alves A . Deciphering promoter hypermethylation of genes encoding for Rassf/Hippo pathway reveals the poor prognostic factor of Rassf2 gene silencing in colon cancers. Cancers. (2021) 13. doi: 10.3390/cancers13235957 34885067 PMC8656858

[37] Whitehurst AW Ram R Shivakumar L Gao B Minna JD White MA . The Rassf1a tumor suppressor restrains anaphase-promoting complex/cyclosome activity during the G1/S phase transition to promote cell cycle progression in human epithelial cells. Mol Cell Biol. (2008) 28:3190–7. doi: 10.1128/mcb.02291-07 18347058 PMC2423179

[38] Shivakumar L Minna J Sakamaki T Pestell R White MA . The Rassf1a tumor suppressor blocks cell cycle progression and inhibits cyclin D1 accumulation. Mol Cell Biol. (2002) 22:4309–18. doi: 10.1128/mcb.22.12.4309-4318.2002 12024041 PMC133879

[39] Thaler S Hähnel PS SChad A Dammann R Schuler M . Rassf1a mediates P21cip1/Waf1-dependent cell cycle arrest and senescence through modulation of the Raf-Mek-Erk pathway and inhibition of Akt. Cancer Res. (2009) 69:1748–57. doi: 10.1158/0008-5472.Can-08-1377 19223555

[40] Liu M Rao H Liu J Li X Feng W Gui L . The histone methyltransferase Setd2 modulates oxidative stress to attenuate experimental colitis. Redox Biol. (2021) 43:102004. doi: 10.1016/j.redox.2021.102004 34020310 PMC8141928

[41] Lai CY Yeh KY Liu BF Chang TM Chang CH Liao YF . Microrna-21 plays multiple oncometabolic roles in colitis-associated carcinoma and colorectal cancer via the Pi3k/Akt, Stat3, and Pdcd4/Tnf-A signaling pathways in zebrafish. Cancers. (2021) 13. doi: 10.3390/cancers13215565 34771727 PMC8583575

[42] Sun F Fu H Liu Q Tie Y Zhu J Xing R . Downregulation of Ccnd1 and Cdk6 by Mir-34a induces cell cycle arrest. FEBS Lett. (2008) 582:1564–8. doi: 10.1016/j.febslet.2008.03.057 18406353

[43] Saito Y Nakaoka T Saito H . Microrna-34a as a therapeutic agent against human cancer. J Clin Med. (2015) 4:1951–9. doi: 10.3390/jcm4111951 26580663 PMC4663478

[44] Lodygin D Tarasov V Epanchintsev A Berking C Knyazeva T Körner H . Inactivation of Mir-34a by aberrant Cpg methylation in multiple types of cancer. Cell Cycle. (2008) 7:2591–9. doi: 10.4161/cc.7.16.6533 18719384

[45] Rapti SM Kontos CK Christodoulou S Papadopoulos IN Scorilas A . Mir-34a overexpression predicts poor prognostic outcome in colorectal adenocarcinoma, independently of clinicopathological factors with established prognostic value. Clin Biochem. (2017) 50:918–24. doi: 10.1016/j.clinbiochem.2017.06.004 28624481

[46] Taniguchi K Karin M . Nf-Kb, inflammation, immunity and cancer: Coming of age. Nat Rev Immunol. (2018) 18:309–24. doi: 10.1038/nri.2017.142 29379212

[47] Wang M Xiao Y Miao J Zhang X Liu M Zhu L . Oxidative stress and inflammation: Drivers of tumorigenesis and therapeutic opportunities. Antioxidants Bsl. (2025) 14. doi: 10.3390/antiox14060735 40563367 PMC12189506

[48] Huang Q Jing Y Xiong L Li L Feng J Cheng J . The interplay between driver mutation and oxidative stress in colorectal cancer: From pathogenesis to therapeutics. J Transl Med. (2025) 23:635. doi: 10.1186/s12967-025-06640-x 40490762 PMC12150591

[49] Pezone A Olivieri F Napoli MV Procopio A Avvedimento EV Gabrielli A . Inflammation and DNA damage: Cause, effect or both. Nat Rev Rheumatol. (2023) 19:200–11. doi: 10.1038/s41584-022-00905-1 36750681

[50] Gasser SM . The double face of base excision repair: Preventing and triggering double-strand breaks. Bioessays. (2026) 48:e70092. doi: 10.1002/bies.70092 41273051 PMC12706135

[51] Choi JE Chung WH . Functional interplay between the oxidative stress response and DNA damage checkpoint signaling for genome maintenance in aerobic organisms. J Microbiol. (2020) 58:81–91. doi: 10.1007/s12275-020-9520-x 31875928

[52] Yates LA Zhang X Burgers PM . DNA damage and replication stress checkpoints. Annu Rev Biochem. (2025) 94:195–221. doi: 10.1146/annurev-biochem-072324-031915 40540755 PMC12893018

[53] Rubin SM Sage J Skotheim JM . Integrating old and new paradigms of G1/S control. Mol Cell. (2020) 80:183–92. doi: 10.1016/j.molcel.2020.08.020 32946743 PMC7582788

[54] Liu K Zheng M Lu R Du J Zhao Q Li Z . The role of Cdc25c in cell cycle regulation and clinical cancer therapy: A systematic review. Cancer Cell Int. (2020) 20:213. doi: 10.1186/s12935-020-01304-w 32518522 PMC7268735

[55] Hankey W McIlhatton MA Ebede K Kennedy B Hancioglu B Zhang J . Mutational mechanisms that activate Wnt signaling and predict outcomes in colorectal cancer patients. Cancer Res. (2018) 78:617–30. doi: 10.1158/0008-5472.Can-17-1357 29212857

[56] Preto A Figueiredo J Velho S Ribeiro AS Soares P Oliveira C . BRAF provides proliferation and survival signals in MSI colorectal carcinoma cells displaying BRAF(V600E) but not KRAS mutations. J Pathol. (2008) 214:320–7. doi: 10.1002/path.2295 18098337

[57] Saleban M Harris EL Poulter JA . D-type cyclins in development and disease. Genes Bsl. (2023) 14. doi: 10.3390/genes14071445 37510349 PMC10378862

[58] Ahmadi Y Faiq T Abolhasani S . Impact of G1 phase kinetics on the acquisition of stemness in cancer cells: The critical role of cyclin D. Mol Biol Rep. (2025) 52:230. doi: 10.1007/s11033-025-10351-3 39951181

[59] Sharma S Bhattacharya S Joshi K Singh S . A shift in focus towards precision oncology, driven by revolutionary nanodiagnostics; revealing mysterious pathways in colorectal carcinogenesis. J Cancer Res Clin Oncol. (2023) 149:16157–77. doi: 10.1007/s00432-023-05331-8 37650995 PMC11797645

[60] Chatila WK Walch H Hechtman JF Moyer SM Sgambati V Faleck DM . Integrated clinical and genomic analysis identifies driver events and molecular evolution of colitis-associated cancers. Nat Commun. (2023) 14:110. doi: 10.1038/s41467-022-35592-9 36611031 PMC9825391

[61] Su H Kang Q Wang H Yin H Duan L Liu Y . Changes in expression of p53 and inflammatory factors in patients with ulcerative colitis. Exp Ther Med. (2019) 17:2451–6. doi: 10.3892/etm.2019.7253 30906432 PMC6425133

[62] Zhou X Santos GS Zhan Y Oliveira MMS Rezaei S Singh M . Mutant p53 gain of function mediates cancer immune escape that is counteracted by APR-246. Br J Cancer. (2022) 127:2060–71. doi: 10.1038/s41416-022-01971-8 36138076 PMC9681866

[63] Chauhan S Jaiswal S Jakhmola V Singh B Bhattacharya S Garg M . Potential role of p53 deregulation in modulating immune responses in human Malignancies: A paradigm to develop immunotherapy. Cancer Lett. (2024) 588:216766. doi: 10.1016/j.canlet.2024.216766 38408603 PMC7615729

[64] Zhang H Xu J Long Y Maimaitijiang A Su Z Li W . Unraveling the guardian: p53's multifaceted role in the DNA damage response and tumor treatment strategies. Int J Mol Sci. (2024) 25. doi: 10.3390/ijms252312928 39684639 PMC11641486

[65] Raveenthiraraj S Awanis G Chieppa M O'Connell AE Sobolewski A . M1 and M2 macrophages differentially regulate colonic crypt renewal. Inflammation Bowel Dis. (2024) 30:1138–50. doi: 10.1093/ibd/izad270 38001043 PMC11219479

[66] Wang Q Li Z Liu K Liu J Chai S Chen G . Activation of the G protein-coupled estrogen receptor prevented the development of acute colitis by protecting the crypt cell. J Pharmacol Exp Ther. (2021) 376:281–93. doi: 10.1124/jpet.120.000216 33318078

[67] Taniguchi K Wu LW Grivennikov SI de Jong PR Lian I Yu FX . A GP130-SRC-YAP module links inflammation to epithelial regeneration. Nature. (2015) 519:57–62. doi: 10.1038/nature14228 25731159 PMC4447318

[68] Jiang L Tian J Yang J Luo R Zhang Y Shao C . P21 regulates Wnt-Notch balance via DREAM/MMB/RB-E2F1 and maintains intestinal stem cell homeostasis. Cell Death Discov. (2024) 10:413. doi: 10.1038/s41420-024-02192-z 39341834 PMC11438959

[69] Xiang J Wang H Tao Q Li W Huang Y Zhang Y . CDK4/6 inhibitor modulating active and quiescent intestinal stem cells for prevention of chemotherapy-induced diarrhea. J Pathol. (2023) 260:235–47. doi: 10.1002/path.6078 36978197

[70] Schmitz ML Kracht M . Cyclin-dependent kinases as coregulators of inflammatory gene expression. Trends Pharmacol Sci. (2016) 37:101–13. doi: 10.1016/j.tips.2015.10.004 26719217

[71] Williams GH Stoeber K . The cell cycle and cancer. J Pathol. (2012) 226:352–64. doi: 10.1002/path.3022 21990031

[72] Gronke K Hernández PP Zimmermann J Klose CSN Kofoed-Branzk M Guendel F . Interleukin-22 protects intestinal stem cells against genotoxic stress. Nature. (2019) 566:249–53. doi: 10.1038/s41586-019-0899-7 30700914 PMC6420091

[73] Fang M Wu HK Pei Y Zhang Y Gao X He Y . E3 ligase MG53 suppresses tumor growth by degrading cyclin D1. Signal Transduct Tgt Ther. (2023) 8:263. doi: 10.1038/s41392-023-01458-9 37414783 PMC10326024

[74] Cole AM Myant K Reed KR Ridgway RA Athineos D Van den Brink GR . Cyclin D2-cyclin-dependent kinase 4/6 is required for efficient proliferation and tumorigenesis following APC loss. Cancer Res. (2010) 70:8149–58. doi: 10.1158/0008-5472.Can-10-0315 20736363 PMC2974087

[75] Lv H Mu Y Zhang C Zhao M Jiang P Xiao S . Comparative analysis of single-cell transcriptome reveals heterogeneity and commonality in the immune microenvironment of colorectal cancer and inflammatory bowel disease. Front Immunol. (2024) 15:1356075. doi: 10.3389/fimmu.2024.1356075 38529274 PMC10961339

[76] Olafsson S McIntyre RE Coorens T Butler T Jung H Robinson PS . Somatic evolution in non-neoplastic IBD-affected colon. Cell. (2020) 182:672–684.e11. doi: 10.1016/j.cell.2020.06.036 32697969 PMC7427325

[77] Choi WT Wen KW Rabinovitch PS Huang D Mattis AN Gill RM . DNA content analysis of colorectal serrated lesions detects an aneuploid subset of inflammatory bowel disease-associated serrated epithelial change and traditional serrated adenomas. Histopathology. (2018) 73:464–72. doi: 10.1111/his.13652 29772067

[78] Manning AL Benes C Dyson NJ . Whole chromosome instability resulting from the synergistic effects of pRB and p53 inactivation. Oncogene. (2014) 33:2487–94. doi: 10.1038/onc.2013.201 23792446 PMC3884049

[79] Zolotovskaia MA Modestov AA Suntsova MV Rachkova AA Koroleva EV Poddubskaya EV . Pan-cancer antagonistic inhibition pattern of ATM-driven G2/M checkpoint pathway vs other DNA repair pathways. DNA Repair Amst. (2023) 123:103448. doi: 10.1016/j.dnarep.2023.103448 36657260

[80] Kanakkanthara A Jeganathan KB Limzerwala JF Baker DJ Hamada M Nam HJ . Cyclin A2 is an RNA binding protein that controls MRE11 mRNA translation. Science. (2016) 353:1549–53. doi: 10.1126/science.aaf7463 27708105 PMC5109925

[81] Guo Y Gabola M Lattanzio R Paul C Pinet V Tang R . Cyclin A2 maintains colon homeostasis and is a prognostic factor in colorectal cancer. J Clin Invest. (2021) 131. doi: 10.1172/jci131517 33332285 PMC7880422

[82] Mirzayans R Andrais B Kumar P Murray D . Significance of wild-type p53 signaling in suppressing apoptosis in response to chemical genotoxic agents: Impact on chemotherapy outcome. Int J Mol Sci. (2017) 18. doi: 10.3390/ijms18050928 28452953 PMC5454841

[83] Tornillo L Lugli A Zlobec I Willi N Glatz K Lehmann F . Prognostic value of cell cycle and apoptosis regulatory proteins in mismatch repair-proficient colorectal cancer: A tissue microarray-based approach. Am J Clin Pathol. (2007) 127:114–23. doi: 10.1309/6rt941w1g6gdehue 17145638

[84] Watson NF Durrant LG Scholefield JH Madjd Z Scrimgeour D Spendlove I . Cytoplasmic expression of p27(Kip1) is associated with a favourable prognosis in colorectal cancer patients. World J Gastroenterol. (2006) 12:6299–304. doi: 10.3748/wjg.v12.i39.6299 17072952 PMC4088137

[85] Guo H Tian T Nan K Wang W . P57: A multifunctional protein in cancer (review). Int J Oncol. (2010) 36:1321–9. doi: 10.3892/ijo_00000617 20428755

[86] Oka T Higa T Sugahara O Koga D Nakayama S Nakayama KI . Ablation of p57+ quiescent cancer stem cells suppresses recurrence after chemotherapy of intestinal tumors. Cancer Res. (2023) 83:1393–409. doi: 10.1158/0008-5472.Can-22-2578 36880956

[87] Baan B Dihal AA Hoff E Bos CL Voorneveld PW Koelink PJ . 5-Aminosalicylic acid inhibits cell cycle progression in a phospholipase D dependent manner in colorectal cancer. Gut. (2012) 61:1708–15. doi: 10.1136/gutjnl-2011-301626 22187071

[88] Ahmed AT Oghenemaro EF Hjazi A Jain V Ahmad I Roopashree R . The significance of STAT3 in colonic diseases: A comprehensive study of pathological roles and therapeutic implications. Cell Biochem Biophys. (2025) 83:4097–120. doi: 10.1007/s12013-025-01816-0 40627320

[89] Pacifico T Stolfi C Tomassini L Luiz-Ferreira A Franzè E Ortenzi A . Rafoxanide negatively modulates STAT3 and NF-KB activity and inflammation-associated colon tumorigenesis. Cancer Sci. (2024) 115:3596–611. doi: 10.1111/cas.16317 39239848 PMC11531958

[90] Wang R Zhou EJ Zhang CY Li LP Yang BB Du TT . Discovery of benzimidazo-2-amino-1,3,4-thiadiazole carboxylate small-molecule STAT3 inhibitors for colorectal carcinoma therapy. Eur J Med Chem. (2026) 306:118588. doi: 10.1016/j.ejmech.2026.118588 41564506

[91] Gong H Chen S Liu S Hu Q Li Y Li Y . Overexpressing lipid raft protein STOML2 modulates the tumor microenvironment via NF-KB signaling in colorectal cancer. Cell Mol Life Sci. (2024) 81:39. doi: 10.1007/s00018-023-05105-y 38214751 PMC10786741

[92] Fassl A Geng Y Sicinski P . CDK4 and CDK6 kinases: From basic science to cancer therapy. Science. (2022) 375:eabc1495. doi: 10.1126/science.abc1495 35025636 PMC9048628

[93] Cao Y Yi Y Han C Shi B . NF-KB signaling pathway in tumor microenvironment. Front Immunol. (2024) 15:1476030. doi: 10.3389/fimmu.2024.1476030 39493763 PMC11530992

[94] Wang S Liu Z Wang L Zhang X . NF-KB signaling pathway, inflammation and colorectal cancer. Cell Mol Immunol. (2009) 6:327–34. doi: 10.1038/cmi.2009.43 19887045 PMC4003215

[95] Guttridge DC Albanese C Reuther JY Pestell RG Baldwin AS . NF-kappaB controls cell growth and differentiation through transcriptional regulation of cyclin D1. Mol Cell Biol. (1999) 19:5785–99. doi: 10.1128/mcb.19.8.5785 10409765 PMC84428

[96] Temby M Boye TL Hoang J Nielsen OH Gubatan J . Kinase signaling in colitis-associated colon cancer and inflammatory bowel disease. Biomolecules. (2023) 13. doi: 10.3390/biom13111620 38002302 PMC10669043

[97] Wu F Sun G Nai Y Shi X Ma Y Cao H . NUP43 promotes PD-L1/NPD-L1/PD-L1 feedback loop via TM4SF1/JAK/STAT3 pathway in colorectal cancer progression and metastasis. Cell Death Discov. (2024) 10:241. doi: 10.1038/s41420-024-02025-z 38762481 PMC11102480

[98] Sun J Du Y Song Q Nan J Guan P Guo J . E2F is required for STAT3-mediated upregulation of cyclin B1 and CDC2 expressions and contributes to G2-M phase transition. Acta Biochim Biophys Sin Shanghai. (2019) 51:313–22. doi: 10.1093/abbs/gmy174 30726872

[99] Deng F Wu Z Zou F Wang S Wang X . The Hippo-YAP/TAZ signaling pathway in intestinal self-renewal and regeneration after injury. Front Cell Dev Biol. (2022) 10:894737. doi: 10.3389/fcell.2022.894737 35927987 PMC9343807

[100] Seo Y Park SY Kim HS Nam JS . The Hippo-Yap signaling as guardian in the pool of intestinal stem cells. Biomedicines. (2020) 8. doi: 10.3390/biomedicines8120560 33271948 PMC7760694

[101] Qin X Cardoso Rodriguez F Sufi J Vlckova P Claus J Tape CJ . An oncogenic phenoscape of colonic stem cell polarization. Cell. (2023) 186:5554–5568.e18. doi: 10.1016/j.cell.2023.11.004 38065080

[102] Mzoughi S Schwarz M Wang X Demircioglu D Ulukaya G Mohammed K . Oncofetal reprogramming drives phenotypic plasticity in WNT-dependent colorectal cancer. Nat Genet. (2025) 57(2):402–12. doi: 10.1038/s41588-024-02058-1 39930084 PMC11821538

[103] Deng F Wu Z Xu M Xia P . Yap activates Stat3 signalling to promote colonic epithelial cell proliferation in DSS-induced colitis and colitis associated cancer. J Inflammation Res. (2022) 15:5471–82. doi: 10.2147/jir.S377077 36164660 PMC9508680

[104] Wang Z Kim SY Tu W Kim J Xu A Yang YM . Extracellular vesicles in fatty liver promote a metastatic tumor microenvironment. Cell Metab. (2023) 35:1209–1226.e13. doi: 10.1016/j.cmet.2023.04.013 37172577 PMC10524732

[105] Cheung P Xiol J Dill MT Yuan WC Panero R Roper J . Regenerative reprogramming of the intestinal stem cell state via Hippo signaling suppresses metastatic colorectal cancer. Cell Stem Cell. (2020) 27:590–604.e9. doi: 10.1016/j.stem.2020.07.003 32730753 PMC10114498

[106] Xu M Xia P Wang S Zou F Wu Z Huang C . Yap inactivated by NF-κB p65, can protect against colonic epithelial cell pyroptosis in ulcerative colitis via transcriptionally regulating NLRP3. Life Sci. (2026) 386:124167. doi: 10.1016/j.lfs.2025.124167 41443466

[107] Deng F Peng L Li Z Tan G Liang E Chen S . Yap triggers the Wnt/β-catenin signalling pathway and promotes enterocyte self-renewal, regeneration and tumorigenesis after DSS-induced injury. Cell Death Dis. (2018) 9:153. doi: 10.1038/s41419-017-0244-8 29396428 PMC5833613

[108] Zhang H Li Z Jiang J Lei Y Xie J Liu Y . SNTB1 regulates colorectal cancer cell proliferation and metastasis through YAP1 and the Wnt/β-catenin pathway. Cell Cycle. (2023) 22:1865–83. doi: 10.1080/15384101.2023.2244778 37592763 PMC10599191

[109] Hoxha S Shepard A Troutman S Diao H Doherty JR Janiszewska M . Yap-mediated recruitment of YY1 and EZH2 represses transcription of key cell-cycle regulators. Cancer Res. (2020) 80:2512–22. doi: 10.1158/0008-5472.Can-19-2415 32409309 PMC7299785

[110] Benzeno S Narla G Allina J Cheng GZ Reeves HL Banck MS . Cyclin-dependent kinase inhibition by the KLF6 tumor suppressor protein through interaction with cyclin D1. Cancer Res. (2004) 64:3885–91. doi: 10.1158/0008-5472.Can-03-2818 15172998

[111] Lang UE Kocabayoglu P Cheng GZ Ghiassi-Nejad Z Muñoz U Vetter D . GSK3β phosphorylation of the KLF6 tumor suppressor promotes its transactivation of p21. Oncogene. (2013) 32:4557–64. doi: 10.1038/onc.2012.457 23085750 PMC3892988

[112] Grivennikov SI Karin M . Dangerous liaisons: Stat3 and NF-kappaB collaboration and crosstalk in cancer. Cytokine Growth Factor Rev. (2010) 21:11–9. doi: 10.1016/j.cytogfr.2009.11.005 20018552 PMC2834864

[113] Wang Q Gao X Yu T Yuan L Dai J Wang W . Regγ controls Hippo signaling and reciprocal NF-κB-Yap regulation to promote colon cancer. Clin Cancer Res. (2018) 24:2015–25. doi: 10.1158/1078-0432.Ccr-17-2986 29437787

[114] Xia P Deng F . Yap regulates intestinal epithelial cell proliferation through activation of Stat3 in DSS-induced colitis and associated cancer. Zhong Nan Da Xue Xue Bao Yi Xue Ban. (2022) 47:1637–45. doi: 10.11817/j.issn.1672-7347.2022.220001 36748373 PMC10930267

[115] Noe O Filipiak L Royfman R Campbell A Lin L Hamouda D . Adenomatous polyposis coli in cancer and therapeutic implications. Oncol Rev. (2021) 15:534. doi: 10.4081/oncol.2021.534 34267890 PMC8256374

[116] van Dekken H Wink JC Vissers KJ Franken PF Ruud Schouten W WC JH . Wnt pathway-related gene expression during Malignant progression in ulcerative colitis. Acta Histochem. (2007) 109:266–72. doi: 10.1016/j.acthis.2007.02.007 17445872

[117] Jang J Jung Y Kim Y Jho EH Yoon Y . LPS-induced inflammatory response is suppressed by Wnt inhibitors, Dickkopf-1 and LGK974. Sci Rep. (2017) 7:41612. doi: 10.1038/srep41612 28128299 PMC5269682

[118] Steinbrecher KA Wilson W Cogswell PC Baldwin AS . Glycogen synthase kinase 3beta functions to specify gene-specific, NF-kappaB-dependent transcription. Mol Cell Biol. (2005) 25:8444–55. doi: 10.1128/mcb.25.19.8444-8455.2005 16166627 PMC1265740

[119] Lee CG Hwang S Gwon SY Park C Jo M Hong JE . Bacteroides fragilis toxin induces intestinal epithelial cell secretion of interleukin-8 by the E-cadherin/β-catenin/NF-κB dependent pathway. Biomedicines. (2022) 10. doi: 10.3390/biomedicines10040827 35453577 PMC9032310

[120] Ibrahem S Al-Ghamdi S Baloch K Muhammad B Fadhil W Jackson D . Stat3 paradoxically stimulates β-catenin expression but inhibits β-catenin function. Int J Exp Pathol. (2014) 95:392–400. doi: 10.1111/iep.12102 25348333 PMC4285465

[121] Ernst M Phesse TJ Thiem S Buchert M . 78: Therapeutic inhibition of gp130/Jak/Stat3-dependent cytokine signaling suppresses Wnt-dependent colon cancer formation. Cytokine. (2013) 63:261. doi: 10.1016/j.cyto.2013.06.081 38826717

[122] Kriz V Korinek V . Wnt, Rspo and Hippo signalling in the intestine and intestinal stem cells. Genes Bsl. (2018) 9. doi: 10.3390/genes9010020 29316729 PMC5793173

[123] Edwards AC Stalnecker CA Jean Morales A Taylor KE Klomp JE Klomp JA . TEAD inhibition overcomes YAP1/TAZ-driven primary and acquired resistance to KRASG12C inhibitors. Cancer Res. (2023) 83:4112–29. doi: 10.1158/0008-5472.Can-23-2994 37934103 PMC10821578

[124] Dattani A Huang T Liddle C Smith A Guo G . Suppression of YAP safeguards human naïve pluripotency. Development. (2022) 149. doi: 10.1242/dev.200988 36398796 PMC9845734

[125] Kim M Kim M Park SJ Lee C Lim DS . Role of angiomotin-like 2 mono-ubiquitination on YAP inhibition. EMBO Rep. (2016) 17:64–78. doi: 10.15252/embr.201540809 26598551 PMC4718409

[126] Wang W Li N Li X Tran MK Han X Chen J . Tankyrase inhibitors target YAP by stabilizing angiomotin family proteins. Cell Rep. (2015) 13:524–32. doi: 10.1016/j.celrep.2015.09.014 26456820 PMC4618173

[127] Hong AW Meng Z Guan KL . The Hippo pathway in intestinal regeneration and disease. Nat Rev Gastroenterol Hepatol. (2016) 13:324–37. doi: 10.1038/nrgastro.2016.59 27147489 PMC5642988

[128] Li J Chen D Shen M . Tumor microenvironment shapes colorectal cancer progression, metastasis, and treatment responses. Front Med Lausanne. (2022) 9:869010. doi: 10.3389/fmed.2022.869010 35402443 PMC8984105

[129] Goenka A Khan F Verma B Sinha P Dmello CC Jogalekar MP . Tumor microenvironment signaling and therapeutics in cancer progression. Cancer Commun Lond. (2023) 43:525–61. doi: 10.1002/cac2.12416 37005490 PMC10174093

[130] Guan L Tang Y Li G Qin Z Li S . Comprehensive analysis of role of cyclin-dependent kinases family members in colorectal cancer. Front Oncol. (2022) 12:921710. doi: 10.3389/fonc.2022.921710 35814446 PMC9258493

[131] S MM . Cyclin-dependent kinases as potential targets for colorectal cancer: Past, present and future. Future Med Chem. (2022) 14:1087–105. doi: 10.4155/fmc-2022-0064 35703127

[132] Zhu Q Wei X Qu Z Lu L Zhang Y Wang H . The roles of cell cycle proteins in regulating the tumor immune microenvironment. Genes Dis. (2025) 13(1):101706. doi: 10.1016/j.gendis.2025.101706 41112524 PMC12528906

[133] Arora L Kalia M Pal D . Role of macrophages in cancer progression and targeted immunotherapies. Adv Protein Chem Struct Biol. (2023) 135:281–311. doi: 10.1016/bs.apcsb.2022.11.010 37061335

[134] Löfroos AB Kadivar M Resic Lindehammer S Marsal J . Colorectal cancer-infiltrating T lymphocytes display a distinct chemokine receptor expression profile. Eur J Med Res. (2017) 22:40. doi: 10.1186/s40001-017-0283-8 29020986 PMC5637168

[135] Fridman WH Pagès F Sautès-Fridman C Galon J . The immune contexture in human tumours: Impact on clinical outcome. Nat Rev Cancer. (2012) 12:298–306. doi: 10.1038/nrc3245 22419253

[136] Li J Stanger BZ . Cell cycle regulation meets tumor immunosuppression. Trends Immunol. (2020) 41:859–63. doi: 10.1016/j.it.2020.07.010 32800703 PMC12118812

[137] Thoma OM Naschberger E Kubánková M Larafa I Kramer V Menchicchi B . P21 prevents the exhaustion of CD4(+) T cells within the antitumor immune response against colorectal cancer. Gastroenterology. (2024) 166:284–297.e11. doi: 10.1053/j.gastro.2023.09.017 37734420

[138] Omer OS Hertweck A Roberts LB Lo JW Clough JN Jackson I . Cyclin-dependent kinase 9 as a potential target for anti-TNF-resistant inflammatory bowel disease. Cell Mol Gastroenterol Hepatol. (2022) 14:625–41. doi: 10.1016/j.jcmgh.2022.05.011 35660024 PMC9356186

[139] Wang J Liu J Tian F Zhan Y Kong D . Cyclin-dependent kinase 9 expression and its association with CD8(+) T cell infiltration in microsatellite-stable colorectal cancer. Oncol Lett. (2019) 18:6046–56. doi: 10.3892/ol.2019.10970 31788079 PMC6865572

[140] Roskoski R . Cyclin-dependent protein serine/threonine kinase inhibitors as anticancer drugs. Pharmacol Res. (2019) 139:471–88. doi: 10.1016/j.phrs.2018.11.035 30508677

[141] Agostinetto E Arecco L de Azambuja E . Adjuvant CDK4/6 inhibitors for early breast cancer: How to choose wisely? Oncol Ther. (2024) 12:19–29. doi: 10.1007/s40487-023-00250-7 37989811 PMC10881905

[142] Wen Y Yang X Li S Huang L Chen J Tan L . Targeting CDK4/6 suppresses colorectal cancer by destabilizing YAP1. MedComm. (2025) 6:e70103. doi: 10.1002/mco2.70103 39968498 PMC11832431

[143] Lee MS Helms TL Feng N Gay J Chang QE Tian F . Efficacy of the combination of MEK and CDK4/6 inhibitors *in vitro* and *in vivo* in KRAS mutant colorectal cancer models. Oncotarget. (2016) 7:39595–608. doi: 10.18632/oncotarget.9153 27167191 PMC5129956

[144] Sorokin AV Kanikarla Marie P Bitner L Syed M Woods M Manyam G . Targeting RAS mutant colorectal cancer with dual inhibition of MEK and CDK4/6. Cancer Res. (2022) 82:3335–44. doi: 10.1158/0008-5472.Can-22-0198 35913398 PMC9478530

[145] Aslam R Richards CE Fay J Hudson L Workman J Lee CL . Synergistic effects of the combination of alpelisib (PI3K inhibitor) and ribociclib (CDK4/6 inhibitor) in preclinical colorectal cancer models. Int J Mol Sci. (2024) 25. doi: 10.3390/ijms252413264 39769028 PMC11676898

[146] Li Z Yin P . Tumor microenvironment diversity and plasticity in cancer multidrug resistance. Biochim Biophys Acta Rev Cancer. (2023) 1878:188997. doi: 10.1016/j.bbcan.2023.188997 37832894

[147] Jiang W He Y He W Zhang X Chen N Li Y . Metastatic sites and lesion numbers cooperated to predict efficacy of PD-1 inhibitor-based combination therapy for patients with metastatic colorectal cancer. Cancer Med. (2023) 12:12482–94. doi: 10.1002/cam4.5959 37081776 PMC10278516

[148] Lin X Kang K Chen P Zeng Z Li G Xiong W . Regulatory mechanisms of PD-1/PD-L1 in cancers. Mol Cancer. (2024) 23:108. doi: 10.1186/s12943-024-02023-w 38762484 PMC11102195

[149] Liu D Xiao H Xiang Y Zhong D Liu Y Wang Y . Strategies to overcome PD-1/PD-L1 blockade resistance: Focusing on combination with immune checkpoint blockades. J Cancer. (2025) 16:3425–49. doi: 10.7150/jca.108163 40861801 PMC12374963

[150] Yi M Niu M Xu L Luo S Wu K . Regulation of Pd-L1 expression in the tumor microenvironment. J Hematol Oncol. (2021) 14:10. doi: 10.1186/s13045-020-01027-5 33413496 PMC7792099

[151] Ribas A . Adaptive immune resistance: How cancer protects from immune attack. Cancer Discov. (2015) 5:915–9. doi: 10.1158/2159-8290.Cd-15-0563 26272491 PMC4560619

[152] Gajewski TF Corrales L Williams J Horton B Sivan A Spranger S . Cancer immunotherapy targets based on understanding the T cell-inflamed versus non-T cell-inflamed tumor microenvironment. Adv Exp Med Biol. (2017) 1036:19–31. doi: 10.1007/978-3-319-67577-0_2 29275462 PMC6693322

[153] Ayers M Lunceford J Nebozhyn M Murphy E Loboda A Kaufman DR . Ifn-γ-related mRNA profile predicts clinical response to Pd-1 blockade. J Clin Invest. (2017) 127:2930–40. doi: 10.1172/jci91190 28650338 PMC5531419

[154] Kalbasi A Ribas A . Tumour-intrinsic resistance to immune checkpoint blockade. Nat Rev Immunol. (2020) 20:25–39. doi: 10.1038/s41577-019-0218-4 31570880 PMC8499690

[155] Zhang J Bu X Wang H Zhu Y Geng Y Nihira NT . Cyclin D-Cdk4 kinase destabilizes Pd-L1 via Cullin 3-Spop to control cancer immune surveillance. Nature. (2018) 553:91–5. doi: 10.1038/nature25015 29160310 PMC5754234

[156] Petroni G Formenti SC Chen-Kiang S Galluzzi L . Immunomodulation by anticancer cell cycle inhibitors. Nat Rev Immunol. (2020) 20:669–79. doi: 10.1038/s41577-020-0300-y 32346095 PMC7584736

[157] Shen J Gong X Ren H Tang X Yu H Tang Y . Identification and validation of Cdk1 as a promising therapeutic target for eriocitrin in colorectal cancer: A combined bioinformatics and experimental approach. BMC Cancer. (2025) 25:76. doi: 10.1186/s12885-025-13448-x 39806333 PMC11731355

[158] Liu S Wu J Lu X Guo C Zheng Q Wang Y . Targeting Cdk12 obviates the Malignant phenotypes of colorectal cancer through the Wnt/β-catenin signaling pathway. Exp Cell Res. (2023) 428:113613. doi: 10.1016/j.yexcr.2023.113613 37100369

[159] Huang C Du R Jia X Liu K Qiao Y Wu Q . Cdk15 promotes colorectal cancer progression via phosphorylating Pak4 and regulating β-catenin/ Mek-Erk signaling pathway. Cell Death Diff. (2022) 29:14–27. doi: 10.1038/s41418-021-00828-6 34262144 PMC8738751

[160] Rahaman MH Lam F Zhong L Teo T Adams J Yu M . Targeting Cdk9 for treatment of colorectal cancer. Mol Oncol. (2019) 13:2178–93. doi: 10.1002/1878-0261.12559 31398271 PMC6763784

[161] Villalona-Calero M Mita M Mita A Federman N Rasco D Spigel D . A first-in-human study of Cdk9 inhibitor Kb-0742 demonstrates evidence of tolerability and clinical activity. Mol Cancer Ther. (2023) 22. doi: 10.1158/1535-7163.TARG-23-B159 36230740

[162] Ortiz-Ruiz MJ Popoola O Mitsopoulos K Te-Poele R Samant RS Box G . Mediator kinase inhibitor selectivity and activity in colorectal cancer. ACS Chem Biol. (2025) 20:1792–804. doi: 10.1021/acschembio.5c00338 40601435 PMC12281475

[163] Sooraj D Sun C Doan A Garama DJ Dannappel MV Zhu D . Med12 and Brd4 cooperate to sustain cancer growth upon loss of mediator kinase. Mol Cell. (2022) 82:123–139.e7. doi: 10.1016/j.molcel.2021.11.015 34910943

[164] Ghosh MK Roy S . Chromosomal instability (CIN) triggers immune evasion and metastatic potential in cancer through rewired STING signalling. Mol BioMed. (2024) 5:4. doi: 10.1186/s43556-023-00166-8 38253764 PMC10803705

[165] Ha CT Tageldein MM Harding SM . The entanglement of DNA damage and pattern recognition receptor signaling. DNA Repair Amst. (2024) 133:103595. doi: 10.1016/j.dnarep.2023.103595 37988925

[166] Samson N Ablasser A . The cGAS-STING pathway and cancer. Nat Cancer. (2022) 3:1452–63. doi: 10.1038/s43018-022-00468-w 36510011

[167] Khorasani M . Role of cGAS-STING in colorectal cancer: A new window for treatment strategies. Cytokine. (2024) 173:156422. doi: 10.1016/j.cyto.2023.156422 37948979

[168] Wang C Gao X Li Y Li C Ma Z Sun D . A molecular subtyping associated with the cGAS-STING pathway provides novel perspectives on the treatment of ulcerative colitis. Sci Rep. (2024) 14:12683. doi: 10.1038/s41598-024-63695-4 38831059 PMC11148070

[169] Li G Zhao X Zheng Z Zhang H Wu Y Shen Y . cGAS-STING pathway mediates activation of dendritic cell sensing of immunogenic tumors. Cell Mol Life Sci. (2024) 81:149. doi: 10.1007/s00018-024-05191-6 38512518 PMC10957617

[170] Liang D Jiang X . When Cdk4/6i meets Gpx4i: Stop dividing to die iron hard. Cell Chem Biol. (2024) 31:187–9. doi: 10.1016/j.chembiol.2024.01.006 38364774 PMC11298777

[171] Lee H Horbath A Kondiparthi L Meena JK Lei G Dasgupta S . Cell cycle arrest induces lipid droplet formation and confers ferroptosis resistance. Nat Commun. (2024) 15:79. doi: 10.1038/s41467-023-44412-7 38167301 PMC10761718

[172] Pleguezuelos-Manzano C Puschhof J Rosendahl Huber A van Hoeck A Wood HM Nomburg J . Mutational signature in colorectal cancer caused by genotoxic Pks(+) E. coli. Nature. (2020) 580:269–73. doi: 10.1038/s41586-020-2080-8 32106218 PMC8142898

[173] Terlouw D Boot A Ducarmon QR Nooij S Suerink M van Leerdam ME . Enrichment of colibactin-associated mutational signatures in unexplained colorectal polyposis patients. BMC Cancer. (2024) 24:104. doi: 10.1186/s12885-024-11849-y 38238650 PMC10797792

[174] Fan Y Li Y Gu X Chen N Chen Y Fang C . Intestinal metabolites in colitis-associated carcinogenesis: Building a bridge between host and microbiome. Chin Med J Engl. (2025) 138:1961–72. doi: 10.1097/cm9.0000000000003430 40287783 PMC12369790

[175] Duizer C de Zoete MR . The role of microbiota-derived metabolites in colorectal cancer. Int J Mol Sci. (2023) 24:8024. doi: 10.3390/ijms24098024 37175726 PMC10178193

[176] Bordonaro M . A model of butyrate activity and resistance in CRC. J Cell Mol Med. (2025) 29:e70656. doi: 10.1111/jcmm.70656 40506777 PMC12162265

[177] Zhang MJ Chan SX Jia ZG Lv C Chen JJ Hong SC . Roles of intestinal stem cells in inflammatory bowel disease pathogenesis. World J Stem Cells. (2025) 17:107639. doi: 10.4252/wjsc.v17.i8.107639 40951705 PMC12427060

[178] Bretou M Sannerud R Escamilla-Ayala A Leroy T Vrancx C Van Acker ZP . Accumulation of APP C-terminal fragments causes endolysosomal dysfunction through the dysregulation of late endosome to lysosome-ER contact sites. Dev Cell. (2024) 59:1571–1592.e9. doi: 10.1016/j.devcel.2024.03.030 38626765

[179] Chakravarti D DePinho RA . Telomere dysfunction as an initiator of inflammation: Clues to an age-old mystery. J Inflammation Bowel Dis Disord. (2021) 6. PMC8516314 34661200

[180] Wu Z Qu J Liu GH . Roles of chromatin and genome instability in cellular senescence and their relevance to ageing and related diseases. Nat Rev Mol Cell Biol. (2024) 25:979–1000. doi: 10.1038/s41580-024-00775-3 39363000

[181] Zhou P Sun L Liu D Liu C Sun L . Long non-coding RNA LincRNA-ROR promotes the progression of colon cancer and holds prognostic value by associating with miR-145. Pathol Oncol Res. (2016) 22:733–40. doi: 10.1007/s12253-016-0061-x 27071407

[182] Ma J Pei J Zhang X Bai X Ding S Dai D . Colon cancer-associated transcript 1 (CCAT1): A potential novel target in cancer therapy. Chin Med J Engl. (2024) 137:2128–30. doi: 10.1097/cm9.0000000000003092 38945528 PMC11374205

[183] Gao H Song X Kang T Yan B Feng L Gao L . Long noncoding RNA CRNDE functions as a competing endogenous RNA to promote metastasis and oxaliplatin resistance by sponging miR-136 in colorectal cancer. Onco Targets Ther. (2017) 10:205–16. doi: 10.2147/ott.S116178 28115855 PMC5221653

[184] Wang C Zhu X Pu C Song X . Upregulated plasmacytoma variant translocation 1 promotes cell proliferation, invasion and metastasis in colorectal cancer. Mol Med Rep. (2018) 17:6598–604. doi: 10.3892/mmr.2018.8669 29512788 PMC5928643

[185] Wu S Dai X Zhu Z Fan D Jiang S Dong Y . Reciprocal regulation of lncRNA MEF and c-Myc drives colorectal cancer tumorigenesis. Neoplasia. (2024) 49:100971. doi: 10.1016/j.neo.2024.100971 38301392 PMC10847691

[186] Ramezani M Shamsabadi FT Shahbazi M . Harnessing the TP53INP1/TP53I3 axis for inhibition of colorectal cancer cell proliferation through MEG3 and Linc-ROR co-expression. Heliyon. (2024) 10:e34075. doi: 10.1016/j.heliyon.2024.e34075 39108882 PMC11301216

[187] Hsieh PF Yu CC Chu PM Hsieh PL . Long non-coding RNA MEG3 in cellular stemness. Int J Mol Sci. (2021) 22. doi: 10.3390/ijms22105348 34069546 PMC8161278

[188] Wan D Wang S Xu Z Zan X Liu F Han Y . PRKAR2A-derived circular RNAs promote the Malignant transformation of colitis and distinguish patients with colitis-associated colorectal cancer. Clin Transl Med. (2022) 12:e683. doi: 10.1002/ctm2.683 35184406 PMC8858608

[189] Hsu CY Almajidi YQ Al-Hakeem MA Alshahrani MY Nabil W Jayakumar SS . Deregulated cell cycle control: The interplay between non-coding RNAs and cyclin-dependent kinases in tumorigenesis. Semin Oncol. (2025) 52:152395. doi: 10.1016/j.seminoncol.2025.152395 40803047

