# Correction: Cell cycle dysregulation: a central hub in colitis-associated colorectal carcinogenesis

**DOI:** 10.3389/fimmu.2026.1940891

**Published:** 2026-08-17

**Authors:** Yuanjie Fu, Ying Wang, Shunjing Wu, Yi Ying, Junxin Li, Yanghuan Ou, Yinying Wang, Li Li, Yueying Wu, Jinyuan Yan, Zhongshan Yang

**Affiliations:** 1 Yunnan Provincial Key Laboratory of Integrated Traditional Chinese and Western Medicine for Chronic Disease in Prevention and Treatment, Yunnan University of Traditional Chinese Medicine, Kunming, Yunnan, China; 2 Yunnan Provincial Department of Education, Engineering Research Center of Classic Formula Regulate Immunity in Chronic Disease Prevention and Treatment, Kunming, Yunnan, China; 3 Artemisinin Research Center, and Institute of Chinese Materia Medica, China Academy of Chinese Medical Sciences, Beijing, China; 4 Center Laboratory of the Second Hospital Affiliated, Kunming Medical University, Kunming, China

**Keywords:** cell cycle, colorectal “inflammation-cancer” transformation, cyclin-dependent kinase inhibitors, Hippo–YAP pathway, oxidative stress

The **Author contributions** statement was erroneously given as “YF: Conceptualization, Project administration, Writing – original draft. YingW: Conceptualization, Project administration, Writing – original draft. YY: Resources, Validation, Writing – review & editing. JL: Formal analysis, Investigation, Writing – review & editing. YO: Formal analysis, Investigation, Writing – review & editing. YinW: Data curation, Funding acquisition, Writing – review & editing. SW: Writing – review & editing. LL: Writing – review & editing. YueW: Conceptualization, Resources, Supervision, Writing – review & editing. JY: Conceptualization, Resources, Supervision, Writing – review & editing. ZY: Conceptualization, Resources, Supervision, Writing – review & editing”.

The correct **Author contributions** statement is “YF: Conceptualization, Project administration, Writing – original draft. YingW: Conceptualization, Project administration, Writing – original draft. SW: Conceptualization, Project administration, Writing – original draft. YiY: Resources, Validation, Writing – review & editing. JL: Formal analysis, Investigation, Writing – review & editing. YO: Formal analysis, Investigation, Writing – review & editing. YW: Data curation, Funding acquisition, Writing – review & editing. LiL: Writing – review & editing. YW: Conceptualization, Resources, Supervision, Writing – review & editing. JY: Conceptualization, Resources, Supervision, Writing – review & editing. ZY: Conceptualization, Resources, Supervision, Writing – review & editing”.

Author Shunjing Wu was erroneously omitted as equal contributing author.

An incorrect number was provided for funder Western Medicine for Chronic Disease in Prevention and Treatment - YPKLS2025-03. The correct number is YPKLS2025-036. The corrected **Funding** statement reads:

“The author(s) declared that financial support was received for this work and/or its publication. This work was supported with funding support from the National Natural Science Foundation of China (82360017) and the Yunnan Provincial Science and Technology Department (202101AZ070001-012,202201AS070084, 202301AZ070001-004, 202407AB110020, 202401AZ070001-025, 202403AC100007); Scientific Research Fund project of Education Department of Yunnan Province (2024Y387, 2026Y0656) Open Research Fund Program of Yunnan Key Laboratory of Integrated Traditional Chinese and Western Medicine for Chronic Disease in Prevention and Treatment (YPKLS2025-036, YPKLS2025-009, YPKLG2024-008, YPKLG2024-007, CWCD2023-030); Institutional Research Project Fund of School of Basic Medical Sciences of Yunnan University of Chinese Medicine (2023CBMS005). Yunnan Provincial University Service Key Industry Science and Technology Project (FWCY-BSPY2024093)”.

The + in “CD8^+^” was not in superscript. A correction has been made to the section **6 Emerging mechanisms linking inflammation to disruption of the cell cycle**, *6.1 cGAS-STING pathway*:

“In the later stage of tumor formation, dendritic cells, upon sensing dsDNA from tumor cells through the cGAS-STING pathway, can mature and efficiently cross-present tumor antigens, thereby activating CD8^+^ T cells and mediating the immune clearance of the tumor (169)”.

The + in “CD4^+^” was not in superscript. A correction has been made to the section **7 Discussion**, paragraph 1:

“The p21 deficiency in CD4^+^ T cells promotes cell exhaustion and tumor growth, and can be reversed by CDK4/6 inhibition, highlighting the importance of considering the immune cell cycle status in treatment design”.

The “m” in “MiR” was written in lowercase. A correction has been made to the section **1 Introduction**, Paragraph 10:

“Since the transcription of MiR-34a is highly dependent on the functional p53, in IBD-CRC, the mutations or loss of function of p53 directly leads to a significant downregulation of miR-34a expression (43)”.

In P21, the letter “p” is written in lowercase. A correction has been made to the section **2 Chronic intestinal inflammation causes abnormal cell cycle through oxidative stress, thereby promoting the occurrence and development of colorectal cancer**, Paragraph 21:

“P21 is regulated by p53 transcription and plays a central role in cell cycle arrest and DNA damage repair. When DNA damage occurs, the activation of p53 induces the expression of p21, thereby inhibiting CDK activity and promoting cell cycle arrest, providing conditions for DNA repair (82)”.

The original version of this article has been updated.

